# Harmonized Peptide Libraries Enable Practical Biofluid Selection for Developing Biomarker Assays

**DOI:** 10.1016/j.mcpro.2025.101086

**Published:** 2025-10-12

**Authors:** Katelyn B. Brusach, Ariana E. Shannon, Alex W. Joyce, Jessica M. Quimby, Brian C. Searle

**Affiliations:** 1 Department of Veterinary Clinical Sciences, The Ohio State University College of Veterinary Medicine, Columbus, Ohio, USA; 2 Pelotonia Institute for Immuno-Oncology, The Ohio State University Comprehensive Cancer Center, Columbus, Ohio, USA; 3 Department of Biomedical Informatics, The Ohio State University Medical Center, Columbus, Ohio, USA

**Keywords:** biofluids, proteomics, urine, serum, plasma, biomarkers, mass spectrometry, parallel reaction monitoring, cats, feline

## Abstract

Selecting the optimal biofluid for accurate biomarker assessment is vital to an informative clinical assay. However, in the initial stages of candidate biomarker discovery, the biologically appropriate biofluid might be unclear. To resolve this dilemma, we demonstrate a mass spectrometry-based workflow where paired urine, plasma, and serum samples are processed in parallel, creating biofluid-specific peptide libraries. These libraries are then harmonized to monitor consistent peptides and transitions, enabling cross-fluid normalization and quantitative comparisons. We also present a reference dataset, “CATalog,” to aid in determining which biofluid to pursue based on protein relative abundance in healthy feline urine, plasma, and serum. Using this workflow and database, we explore the interchangeability of blood biofluid proteins compared to urinary proteins relating to sample processing, relative protein quantification, and clinical application. Our results suggest that when processed correctly, urine could sometimes represent blood biofluid proteins without requiring venipuncture or sample depletion of highly abundant proteins.

## Highlights

• Harmonized libraries enable transition assessment and quantification across matrices.
• CATalog communicates protein relative intensities for informed biofluid selection.
• Biofluid selection may impact protein measurement quality and diagnostic assay impact.
• Urine proteins can represent blood without requiring venipuncture or sample depletion.

## Graphical Abstract

**Figure:**
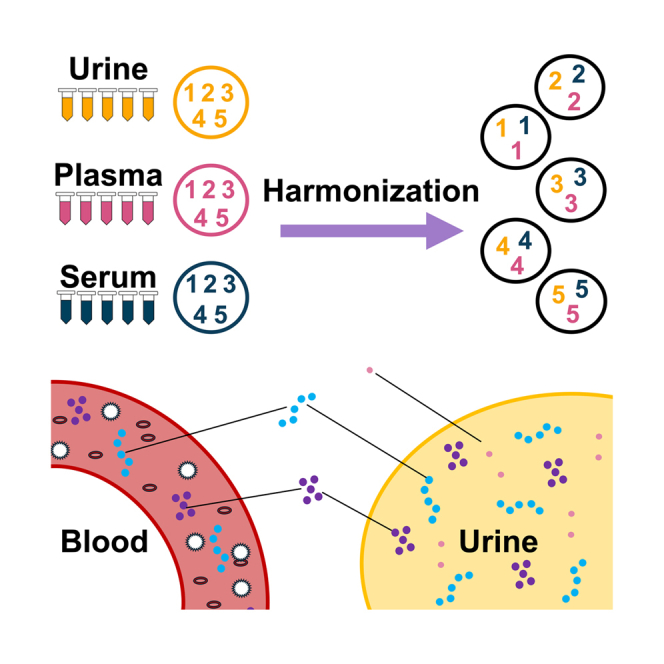

## In Brief

Selecting the optimal biofluid for disease relevance, protein detection, and accessibility is vital for meaningful assays. Biomarker candidates often appear in multiple biofluids, complicating follow-up assessment. Here we present a method to harmonize quantification across groups of samples, enabling us to compare protein abundances between plasma, serum, and urine relative to their dynamic ranges. Our data reveals that biofluid selection can impact protein measurement quality and should be considered before clinical assay development.

Building efficient bridges between biomarker candidate discovery and clinical assay development is vital to achieving rapid advancements in medicine (1). Improvements in mass spectrometry data acquisition and bioinformatics tools enable proteomics to identify and profile novel biomarker candidates more quickly and thoroughly. Biomarkers are ideally found in minimally invasive or noninvasive biomaterials, which is why biofluids such as blood and urine are popular targets for clinical assay development (1, 2). Additionally, blood and urine offer highly informative pathophysiology regarding health and disease due to their direct tissue and organ involvement (3). While biofluids are traditionally a challenging medium to work with due to their large dynamic range and complex matrices, advancements in sample preparation have improved our ability to make both reproducible and meaningful measurements in these matrices (4, 5).

Clinical assays are crucial in diagnosis, prognosis, and monitoring the progression of diseases. Ideally, a good clinical assay is built on reliable proteins that can indicate a pathophysiological state, a process that involves biomarker discovery, analytical verification, and clinical validation (6). However, there is a significant difference in methodology between candidate generation and biomarker validation (7). For the initial candidate generation stage, global proteomic methods utilizing mass spectrometry are able to obtain large quantities of data for researchers to mine for potential biomarker candidates. This is often executed through the use of differential expression analysis between healthy and diseased proteomes (8). Mass spectrometry is also useful for analytical verification, as it can both precisely detect and quantify potential biomarker candidates. When properly validated, ELISAs are excellent at processing a few biomarkers of interest in a large number of samples (9). That said, high-quality ELISAs are not always available for target proteins, and developing a validated ELISA assay is both time-consuming and expensive, costing hundreds of thousands of dollars and taking years to develop (9, 10, 11). Therefore, targeted mass-spectrometry-based proteomics (12, 13) using parallel reaction monitoring (PRM) (14) can be better for the analytical verification of potential biomarker candidates due to the high sensitivity and specificity of high-resolution mass analyzers (15). With advancements in methodologies (16, 17), mass spectrometry is better equipped to bridge the gap between discovery and rigorous analytical verification (18). Despite an abundance of publications on untargeted discovery, targeted verification, and targeted clinical validation, these approaches are not frequently streamlined to be implemented together (19).

Biofluids offer a minimally invasive, or even noninvasive, way of measuring pathophysiological states without requiring tissue samples due to their direct organ involvement. Blood and urine are two of the most promising and popular biofluids for biomarker candidate discovery (19, 20). Due to the differences between blood and urine matrices, proteomic studies that measure urine and blood biofluids typically process them separately (21, 22, 23) and measure different proteins in each. As a result, protein measurements across biofluids are not well characterized, which hinders assay development by assuming one biofluid is better than another without considering protein performance in other matrices. This is especially true in novel biomarker candidates, where the literature on which biofluid to pursue is lacking. A well-established protocol capable of quantifying urine and blood proteins in parallel would allow for optimal biofluid selection when developing an assay.

Due to their differences in matrices, blood and urine are typically prepared differently for mass spectrometry analysis. Approximately 60% of blood proteins are albumin, which shadows the smaller, less abundant proteins (5, 24, 25). Treating blood with strong acids, such as perchloric acid (26), preferentially precipitates albumin and globulins so that smaller, less abundant proteins can be more easily detected (27). For plasma and serum, perchloric acid precipitation is a robustly reproducible approach to improve proteomic depth (28, 29). In contrast, albumin in urine is naturally depleted by the kidneys, so additional depletion is typically not performed. In contrast to blood, the challenge with urine is the low and highly variable protein concentration, which can complicate reproducibility. Using organic solvents, such as acetone, to concentrate and clean the urine helps mitigate this obstacle (30). In blood, both acetone precipitation and perchloric acid depletion can be effective at producing rich proteomes (31). In this work, we sought to acquire a representative proteome of blood and urine biofluids through matrix-appropriate sample cleanup prior to parallel digestion, acquisition, and computational harmonization.

Given the complex nature of mass spectrometry data, interactive and accessible resources are crucial to rapidly and effectively communicating copious quantities of data. Bioinformatic databases that curate biomarker data with user-friendly, interactive summaries are essential tools for organizing preliminary proteomics data. Since the establishment of PepBank, the first searchable archive for peptide sequences, over a dozen databases have been curated (32). Some of these databases focus specifically on therapeutic targets and response biomarkers, including ResMarkerDB (33) and SATPdb (34), while other databases are disease-focused, such as OncoMX (35), Colorectal Cancer Biomarker Database (36), Cancer Peptidome Database of bioFluids (37), and Infectious Diseases Biomarker Database (38). Additionally, protein biomarker databases are typically specific to one biofluid, like urinary protein biomarker database (39), which are specific to urinary proteins. Other databases focus on mass spectrometry data output, such as PeptideAtlas (40), Panorama (41), PRIDE (42), and the mass spectrometry interactive virtual environment (43). PeptideAtlas has even developed subset databases in several nonmodel organisms such as chickens (44), horses (45), pigs (46), cows (47), honey bees (48), and even ticks (49). Overall, these types of databases can be a useful resource for streamlining biomarker discovery by supplying preliminary data in an accessible manner. However, while studies utilizing biofluids are increasingly common, there are no databases that quantitatively compare the same protein across different biofluids.

Selecting the optimal biofluid for disease relevance, ease of protein detection, and sample accessibility is vital to developing a meaningful assay. Often, the literature suggests that biomarker candidates can be monitored in multiple biofluids, complicating follow-up assessments. We propose that database resources could help clinical researchers test discovery-phase biomarker candidates by giving empirical evidence based on relative abundance and consistency. In this study, we measure proteins in paired feline urine, serum, and plasma samples in parallel to allow for direct relative intensity comparison. We also present “CATalog,” a bioinformatics tool that aids in the decision of which biofluid to pursue regarding a selected protein of interest. We then demonstrate the use of this tool as part of a targeted mass spectrometry-based workflow to quantify proteins across biofluids in a chronic kidney disease (CKD) cohort.

## Experimental Procedures

### Animals

To generate the global feline biofluid proteome, eight client-owned healthy cats (Supplementary Table S1*A*) were enrolled through The Ohio State University Veterinary Medical Center. The use of all cats in this study was approved by The Ohio State University International Animal Care and Use Committee (International Animal Care and Use Committee #2020A00000042). The healthy cats were within an age range of 1 to 11 years, with four female and four male cats, all of which were neutered. Prior to enrollment, cats were screened with a complete blood count, serum biochemistry, total thyroxine, urinalysis, blood pressure, and a physical exam to confirm health status. Those with comorbidities were excluded from the study.

The targeted validation assay included five of the eight healthy cats (age range 3–8, three spayed females and two neutered male) in addition to five CKD cats (Supplementary Table S1*B*). Of the CKD cats, there were four neutered males and one spayed female. The age range of these cats was 6 to 20 years. To confirm disease status and International Renal Interest Society staging, cats were screened with bloodwork, urinalysis, and physical exam prior to enrollment. Utilizing their entire medical history, CKD cats were International Renal Interest Society staged (four Stage II cats, one Stage IV cat) based on the International Society of Feline Medicine Consensus Guidelines on the Diagnosis and Management of Feline CKD (50). Those with comorbidities were not enrolled in the study.

### Sample Collection

Paired urine, serum, and plasma samples were collected from each cat. Urine was collected *via* cystocentesis, and samples were spun down at 400 × RCF for 15 min at 4 °C. 500 μl of supernatant was removed and distributed into a cryovial with 10 μl of protease inhibitor (Protease Inhibitor Cocktail, Promega). Blood for serum was collected in a red top tube and was allowed to clot before being spun down at 1500 RCF for 15 min. Serum was removed and transferred to a fresh cryovial with protease inhibitor for storage. Blood for plasma was collected in a purple top EDTA-treated tube before being spun down at 1500 × RCF for 15 min. Plasma was removed and transferred to a cryovial with protease inhibitor. All samples were stored at −80 °C.

### Sample Processing

Samples were randomly distributed into three batches with paired samples blocked together in each batch. Three pooled standards for each biofluid were used to assess any batch effect resulting from sample preparation. Prior to each digestion, each sample was preprocessed to decrease proteome dynamic range by depleting high abundant proteins. However, urine was processed slightly differently compared to serum and plasma. For urine, methods were slightly modified from previously published studies (30, 51). In brief, urine was combined with acetone at a 1:4 volumetric ratio. Each sample was inverted several times, then stored at −20 °C for 16 h. After precipitation, samples were spun down at 12,000 × RCF for 40 min at 4 °C. The supernatant was promptly discarded, and precipitated protein pellets were air dried. Serum and plasma samples were diluted with liquid chromatography mass spectrometry grade water and treated with perchloric acid to a 1:9 ratio of biofluid to solvent (28). Samples were vigorously agitated by inverting rapidly for 10 s, followed by centrifugation at 3200 RCF for 60 min at 4 °C. The protein supernatant was collected, mixed with 1% TFA, and then loaded onto an equilibrated hydrophilic-lipophilic balanced column (Waters, Milford, MA) (conditioned with methanol and equilibrated with 0.1% TFA). Each sample was washed thrice with 0.1% TFA before eluting with 0.1% TFA in 90% acetonitrile. Plasma and serum samples were dried overnight in a SpeedVac. Urine samples were resuspended in 8 M urea in 50 mM tetraethylammonium bromide (TEAB), while serum and plasma samples were resuspended in 5% SDS in 50 mM TEAB.

All samples were measured using Pierce bicinchoninic acid kit (Pierce, Waltham, MA), according to the manufacturer’s instructions. Protein concentration was normalized for all samples to have 100 μg of starting material. All samples were reduced with an excess of 1M DTT for a final concentration of 20 mM DTT, then incubated at 55 °C for 15 min. Proteins were alkylated with 500 mM iodoacetamide for a final concentration of 40 mM iodoacetamide and incubated at room temperature in the dark for 10 min. The alkylation was quenched with 1 M DTT for a final concentration of 20 mM DTT. Next, 27.5% phosphoric acid in water was added to each sample to acidify to a pH below 5. Samples were vortexed before mixing with binding buffer, 100 mM TEAB in 90% methanol, at a 1:7 ratio of protein to buffer, and loaded onto S-Trap columns by centrifuging at 4000 RCF for 1 min. Once trapped, proteins were washed five times with 200 μl of binding buffer followed by centrifugation. Samples were digested on the S-Trap with 1:10 ratio of trypsin for 2 h at 47 °C. Peptides were eluted from the column with 40 μl of 50 mM TEAB in water, 40 μl of 0.2% formic acid in water, and 50% acetonitrile in water. Peptides were dried down with a SpeedVac. Samples were stored at −80 °C until acquisition.

### Global Instrument Analysis

Samples were analyzed using an Easy-nLC1200 coupled to an Orbitrap Exploris 480. All samples were run on the same EasySpray C18 Column (25 cm in length, 75 μm inner diameter, 2 μm packing) column with a trap column (2 cm in length, 75 μm inner diameter, 3 μm packing). Samples were separated with water in 0.1% formic acid (Solvent A) and 80% acetonitrile in 0.1% formic acid (Solvent B) over a 90 min gradient. The gradient started at 2% B for 0 to 5 min, then gradually increased over 5 min to 8% B, with the next increase over 75 min to 28% B, before increasing over 10 min to 44% B, and finally increased to 100% B over 5 min before washing at 100% B for 10 min. Chromatogram libraries for plasma (pool of eight bioreplicates), serum (pool of eight bioreplicates), and urine (pool of eight bioreplicates) were generated for each biofluid using 4 *m/z* wide, staggered isolation windows to deeply profile the proteomes (52). Each bioreplicate was acquired with wide-window data-independent acquisition (DIA) using 16 *m/z* wide, staggered isolated windows, and then processed using EncyclopeDIA (version 2.12.30; https://bitbucket.org/searleb/encyclopedia/) as described below.

### Generating Harmonized Libraries Using EncyclopeDIA

The pooled urine, plasma, and serum Thermo .RAW files were converted to .mzML files using MSConvert (version 3.0.23064; https://proteowizard.sourceforge.io/) as outlined by Pino *et al* (53). Following the GPF-DIA chromatogram library approach, each pooled sample was analyzed as six RAW files, which were each combined into single urine, plasma, and serum spectrum files (DIA) using EncyclopeDIA (52) (version 2.12.30). Individual urine, plasma, and serum files were searched against a Prosit-generated feline library built from all tryptic peptides in a feline Uniport FASTA (May 12, 2023; 51,831 entries). For the tryptic peptides identified, a maximum of two missed cleavages was allowed. Searches considered fixed cysteine carbamidomethylation and no variable modifications. EncyclopeDIA was set to normal target/decoy approach, CID/HCD fragmentation, and all mass tolerances were set to 10.0 PPM. The number of quantitative ions was set to five and the minimum number of quantitative ions was set to three.

After searching each biofluid, individual chromatogram library files were aligned to extract harmonized libraries using a new library management option in EncyclopeDIA called “Extract sample-specific libraries from ELIB”. In this approach, peptides are globally filtered to a 1% false-discovery rate (FDR) level to statistically control for errors across all library files (54). This approach assigned globally identified peptides, where the fragmentation pattern for searching was selected based on the best-scoring biofluid, but maintaining the biofluid-specific retention times for identified peptides. Kernel density estimate alignment (55) and match-between-runs was used to assign retention times for peptides that were not identified in every biofluid.

Lastly, fragment ions used in quantification are preselected to maintain consistent quantification across batches. Rather than choosing the most abundant ions, this approach identifies the ions that correlate best with peak shape across all biofluids in the anticipation that interference in each chromatogram library represents interference in each wide-window DIA experiment. One advantage of this approach is that, due to the changing matrix underlying peptides in each biofluid, fragment ion interference is more easily observed by comparing peak shape in multiple biofluids. Since wide-window measurements are made using wider windows, the windowing scheme for a wide-window DIA run is used to identify situations where co-eluting peptides produce fragment ions that are potentially co-measured due to wider precursor isolation windows. This method results in three harmonized libraries with global fragmentation patterns chosen from the best-performing biofluid for each peptide, biofluid-specific retention times, and protein quantifications based on reliable quantitative ions across all samples.

### Data Processing of Urine, Serum, and Plasma Samples

Bioreplicate Thermo .RAW files were converted to .mzML files using MSConvert (version 3.0.23064). Grouped biofluid files were then uploaded into EncyclopeDIA (version 2.12.30) and searched against their designated harmonized library (urine samples were run against a urine-specific library, serum samples were run against a serum-specific library, and plasma samples were run against a plasma-specific library). Along with individual quant reports for each biofluid, a quant report was also generated with all single injections (eight of serum, eight of plasma, and eight of urine) of all three biofluids run against the pooled biofluid library to easily compare samples in a single quantitative file. A maximum of two missed cleavages was permitted, and searches considered fixed cysteine carbamidomethylation and no variable modifications. A mass tolerance of 10 ppm was considered for precursor and fragment ions, and peptides were filtered at 1% FDR. For quantitation, the number of quantitative ions was set to five, and the minimum number of quantitative ions was set to three.

### CATalog, a Proteomic Biofluid Database

CATalog is written in R using the Shiny framework. The core database consists of two files (.csv): a background file containing the relative intensities from each individual biofluid sample (24 totals) and a higher-level summarization file. Additional.csv files include a GO gene ontology (GO) dataset for *Felis catus* (taxon id 9685) with the fields “biological process”, “cellular compartment”, and “molecular function”, and a ‘deltas’ file that contains the absolute differences between sample intensity for each protein. For each protein, the “top” biofluid(s) are marked if they produce an average signal within 50% of the most intense biofluid signal.

The interface is built using four main tables/figures with a sidebar that facilitates database exploration. Protein relative abundances in urine, plasma, and serum are summarized in the primary table, where protein-specific intensities are summarized using a boxplot. Optional annotations of each boxplot point correspond with a table denoting the age, body condition score (BCS), and sex of each cat utilized in the database. GO metadata of the selected protein is also summarized below the main table. Finally, users can filter proteins based on protein name, accession number, GO metadata, and/or the highest biofluid, and can be further restricted to exclude cats of a certain age, BCS, or sex. Results from one or multiple proteins can be downloaded in an Excel file using the “Shopping Cart” feature.

### Targeted Parallel Reaction Monitoring Assay

Using CATalog, 10 relevant protein biomarker candidates (Supplementary Table S2) were selected for a Tier 3 (56) targeted PRM assay. A pooled sample of serum, urine, and plasma was run prior to the experiment to determine retention time windows. EncyclopeDIA was used to schedule the PRM assay using the PRM Scheduler feature (57). Within the PRM Scheduler, the biofluid-specific harmonized library (described above) was set as the library, an ELIB from the recent DIA injection was used for retention time schedule, five maximum peptides per protein were specified, an assay density of eight scans/second, 10 min retention time window sizes, and a 0.5 *m/z* isolation target offset was set. The target protein accessions of the 10 selected proteins were specified, as well as a list of 40 peptides that were manually validated of having low interference from the chromatogram library. Samples were analyzed using the same chromatography setup as described for the global acquisition. Peptides were resuspended in 2% acetonitrile 0.1% formic acid and 1 μg was loaded onto the column per injection. Isolation windows for PRM were set to 60k resolution at 2 *m/z* width. Ion injection times and windows are listed in Supplementary Table S3.

Skyline (58, 59) (Version 23.1.0.380; https://skyline.ms/home/software/Skyline/project-begin.view) was used to process and export data results. Within Skyline transition settings, peptide settings were set for a tryptic Trypsin [KR|P] as the digestion enzyme with a maximum of two missed cleavages. Background proteome was set to a FASTA list with all target proteins; peptide uniqueness was enforced on the protein level. No retention time predictor was used; however, measured retention times were used when present. Peptides were filtered between a minimum length of seven and a maximum length of 40. Only carbamidomethyl structural modifications on cystines were considered, with three maximum variable modifications. For transition settings, predicted precursor masses and product ion masses were set to monoisotopic. For filtering transients, precursor charges two and three were considered, ion charges of one and two were considered, and ion types y, b, and p were considered. Product ion selection was set from ion three to the last ion, and all matching transitions were selected. For library transition settings, the ion match tolerance was set to 0.05 *m/z*, and if a library spectrum was available, the most intense ion was picked. A minimum of six product ions and a maximum of nine product ions were picked from filtered ion charges and types. For instrument settings, the mass range was set from a minimum of 400 *m/z* to a maximum of 1500 *m/z* with dynamic min product *m/z* selected. The method match tolerance was set to 0.55 *m/z* to account for the 0.5 *m/z* shift for scheduled peptides within the PRM. Triggered chromatogram acquisition was checked. Under full scan settings, MS1 spectra were filtered to three centroided peaks, to a 10 ppm mass accuracy. MS2 filtering was set to PRM for the acquisition method for centroided data within 10 ppm. Retention time filtering was used to select only scans within 5 min of MS/MS IDs. The library was output from EncyclopeDIA’s PRM scheduling feature, which was then converted to a BLIB in EncyclopeDIA before uploading to Skyline (Version 23.1.0.380). All samples were manually integrated, and peptides that did not have a distinguishable chromatogram were eliminated from the assay.

### Experimental Design and Statistical Rationale

Eight biological replicates of paired plasma, serum, and urine were collected to ensure adequate statistical power for global proteomics analysis. For the targeted experiment, bioreplicates of paired plasma, serum, and urine samples from five diseased and five healthy cats were measured. While no prior statistical power calculations were performed, the sample sizes for this study were based on sample availability and study feasibility. Considerations were taken to replicate sample numbers that are consistent with similar studies in the field.

To limit batch effects and retention time shifts between biofluids, samples were block randomized prior to sample preparation and again prior to data acquisition. Blood and urine sample types were analyzed as two separate internal blocks per batch to avoid carryover and retention time fluctuations caused by changing matrices. Pooled standards of urine, serum, and plasma were included in each randomized block to measure variation between batches and biofluids. Furthermore, after a global quantitative measurement was generated using EncyclopeDIA, relative intensities were normalized to the average log_2_ intensity prior to further data analysis. We used a two-sided *t* test to compare diseased *versus* healthy samples in the targeted experiment. This test was appropriate given the independence between the two groups, assumptions of approximate normality, and equality of variance.

## Results and Discussion

Over the past 2 decades, the use of mass spectrometry for biomarker discovery, verification, and validation has become increasingly popular with the advancement of high-resolution mass analyzers (56). Biofluids are a minimally to noninvasive source of pathophysiological information due to their direct involvement with organs. However, there are limited resources that offer information regarding biofluid interchangeability or the advantage of one biofluid over another. The objectives of this study were to 1) present a methodology where biofluid proteins could be directly compared using mass spectrometry, 2) develop a database summarizing the measured intensities of proteins detected in urine, plasma, and serum, and 3) demonstrate how the database can inform assay development in a disease model.

### Considerations in Processing Biofluids in Parallel

Biofluids inherently have different biological matrices. One of the major challenges in quantifying proteins across biofluids is normalizing the samples so that they can be meaningfully compared. We attacked this problem in several ways. Prior to any sample processing, we ensured that the blood, urine, and plasma samples were paired so that there was direct patient-to-patient comparability. Since paired human control samples are challenging to acquire, and mice produce very low volumes of urine, for this pilot data we utilized paired feline samples. Additionally, we limited protocol deviations between biofluids in the preprocessing steps, which preserves the nature of the biofluid while simultaneously decreasing batch effect by processing them in parallel. Finally, we developed a new approach to harmonize across libraries for parallel searching and comparable quantification.

**Fig. 1:**
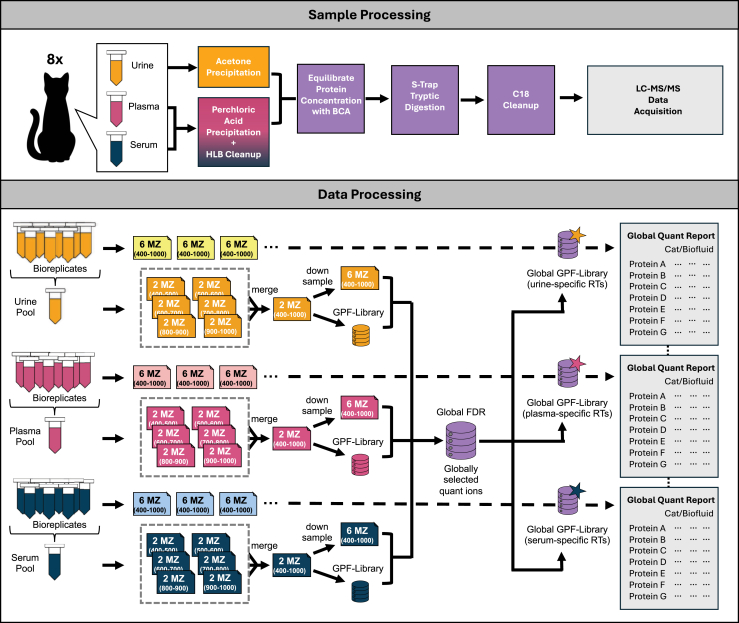
**Schematic of parallel biofluid processing and analysis.** The *top panel* depicts wet-lab sample preparation and data acquisition (Sample Processing) and the *bottom panel* describes the computational harmonization (Data Processing). *Top Panel*: Paired urine, plasma, and serum were collected from eight healthy cats. Urine samples (*yellow*) were precipitated with acetone, while plasma (*pink*) and serum (*blue*) were treated with perchloric acid and an hydrophilic-lipophilic balanced cleanup. Samples processing continued in parallel (*purple*) where samples were adjusted to an equal protein concentration, digested, and then cleaned before data acquisition (*gray*). *Bottom Panel*: Workflow for creating harmonized libraries using globally-selected quantitative peptides and fragment ions. Sample pools of urine (*yellow*), plasma (*pink*), and serum (*blue*) were utilized to make three separate DIA-GPF chromatogram libraries. Fragment ions were checked using a down-sampling approach for potential interference in each biofluid before being marked for downstream quantitative analysis, allowing the library to contain fragment ions marked either as quantitatively harmonized or for detection-only. The libraries were then merged into a harmonized library (*purple*) (further filtered at 1% false-discovery rate globally) before being recalibrated with retention times specific to each biofluid matrix. Searching these libraries created a harmonized quantitative output (*gray*) measuring the same peptides with the same quantitative fragment ions across all biofluids.

Urine is commonly solvent precipitated to remove salts and concentrate proteins, given the varying protein concentrations in different urine samples. Conversely, serum and plasma benefit from an abundant protein depletion step. For this work, we chose to process urine with acetone precipitation and process the blood-based samples with perchloric acid-based depletion and pre-digestion hydrophilic-lipophilic balanced cleanup to remove salts (Fig. 1). After the initial cleanup and resuspension, samples were processed in parallel. Concerned about variation introduced by the initial cleanup, we cross-compared protocols and found that the different sample preparations did not create significant bias in relative protein counts (Supplementary Table S4) or in protein overlap (Supplementary Fig. S1) with one exception: the perchloric acid precipitation negatively affected urine proteomes. As such, we performed matrix-specific sample cleanup and then performed parallel digestion, acquisition, and computational harmonization to assess the similarities and differences of optimally prepared biofluids. Even without depletion, urine identified more proteins than the blood biofluids combined (1920 proteins in urine, 680 proteins in plasma, and 726 proteins in serum). Essentially, healthy kidneys perform a “natural depletion” in which they filter large and highly abundant proteins, such as albumin, a process that is manually required to improve coverage depth in serum and plasma. This allows urine to be rich in small blood-based proteins without requiring extensive sample preparation. Urine is expected to contain approximately 2000 proteins (60); therefore, our findings show a robust overview of the urinary proteome.

**Fig. 2:**
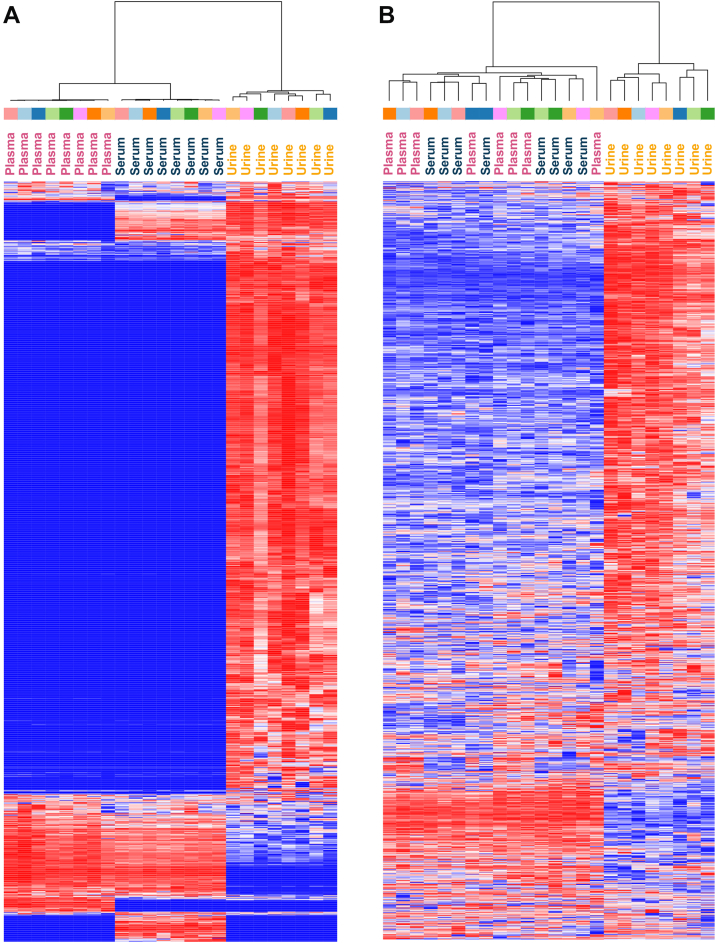
Demonstration of the effects of peptide library harmonization. Heatmap showing paired plasma, serum, and urine protein relative intensities without harmonization (*A*) and after harmonization (*B*). *Color blocks* above biofluid labels indicate which bioreplicate the sample originated from. Without harmonization, all biofluids cluster distinctly from each other. After harmonization, while urine remains separated from serum and plasma, clustering generally groups an individual’s serum and plasma closer together.

While the sample preparation allowed the samples to be compared from a methodological standpoint, it was important that we handle the data with batch specific considerations. Differences in retention times and interference can inhibit direct comparison between biofluids or batches. To test if our sample preparation required further statistical correction from matrix effects, we evaluated the retention time differences between plasma, serum, and urine. After correction, we found that retention times from urine, serum, and plasma showed limited biofluid matrix effects (Supplementary Fig. S2). To additionally correct this, we developed a new EncyclopeDIA feature that creates harmonized libraries (Fig. 1), enabling trustworthy quantification across biofluids. Each biofluid-specific harmonized library contains the full set of peptides assessed across biofluids using global FDR, but with retention times calibrated specifically to each biological matrix. Importantly, potential quantitative fragment ions are checked and selected using a down-sampling approach for potential interference caused by shifting retention times in each biofluid before being marked for downstream analysis. This creates a harmonized quantitative output measuring the same peptides with the same quantitative fragment ions across all biofluids. Briefly, this approach assesses global FDR (across biofluids) as part of library generation but maintains biofluid-specific retention time alignment. In our methodology, we downsample the GPF-DIA datasets used to create the original biofluid libraries and check for potential interferences from other peptides in wide-window files. This lets us choose the best transitions that are interference-free in all libraries at the same time. One advantage of harmonizing libraries together is that our approach lets us choose the best biofluid for each peptide to assess fragmentation patterns for peptide detection. Additionally, by comparing across biofluids, quantitative fragment ions (not affecting detection) are preselected depending on fragment consistency across all matrices. This workflow enables us to process data collected from all three biofluids using the same globally-selected library of peptides, but retaining biofluid-specific retention times. The differences between the samples preand postharmonization are depicted in Figure 2, where harmonization greatly improves the consistency of proteins detected across the three biofluids compared to when processing them individually. Without harmonization (Fig. 2*A*), all biofluids cluster distinctly from each other. After harmonization (Fig. 2*B*), while urine remains separated from serum and plasma, clustering generally groups an individual’s serum and plasma closer together.

### Comparing Matrix, Protein, and Quantitative Surrogacy Between Biofluids

While our methodology successfully identified proteins in urine, plasma, and serum, we sought to further evaluate the matrix, protein, and quantitative interchangeability of biofluids. We approached this question aiming to evaluate if urine proteins could be measured in blood, or vice versa, depending on clinical applicability, and to observe how protein relative abundances compared quantitatively between biofluids. Prior to these comparisons, we evaluated any possible cofounding variables, including age, sex, and BCS, using a hierarchical clustering (Supplementary Fig. S3). There was no visible clustering of proteins according to sex, age, or body score. The lack of difference between sexes is likely attributed to all cats being neutered. Additionally, because all samples were paired, there was concern that matched samples (i.e., paired samples of the same cat) would cluster together. However, we did not observe any clustering based on sample ID (Supplementary Fig. S3), indicating that there are no significant confounding variables to consider when comparing matrices. Urine showed higher relative intensities in many of the proteins compared to either blood biofluid, as well as visible clustering compared to serum and plasma.

**Fig. 3:**
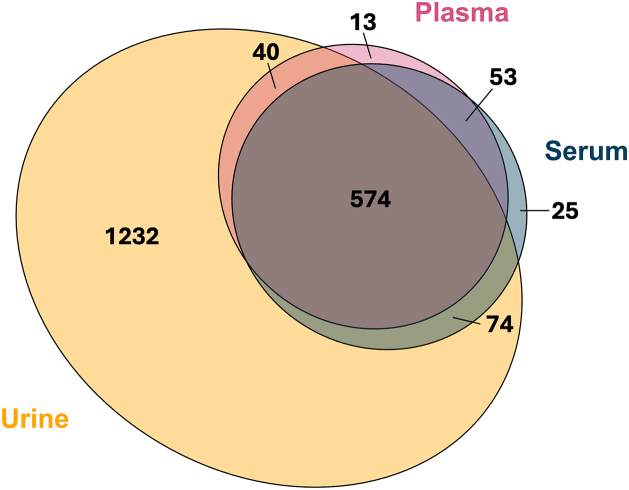
**Venn diagram showing the overlap between 1920 urinary (*yellow*), 680 plasma (*pink*), and 726 serum (*blue*) proteins**.

Of the proteins identified in urine, plasma, and serum, 574 proteins were quantified across all three biofluids (Fig. 3). These overlapping proteins are particularly exciting, as they allow for deeper understanding of disease processes by measuring how the protein abundance changes before and after interacting with the kidney and allowing for optimal biofluid selection based on the assay needs. For example, if one biofluid is easier to obtain or process, or if a protein needs to be added to an assay already reliant on a particular biofluid. Of the shared proteins, 75 overlapped between only serum and plasma, 77 overlapped between only urine and serum, 140 overlapped between only urine and plasma, and 418 proteins were found in all three (Fig. 3). We also considered proteins unique to each biofluid, as these are also important to consider if specifically interested in one biofluid. We found 13 proteins specific to plasma, 25 proteins specific to serum, and 1232 proteins specific to urine (Fig. 3). The large number of urine-specific proteins increases our previously stated excitement of urine as a biofluid to target for biomarker candidates.

Having observed visual clustering of urine compared to serum and plasma, we then applied a PCA analysis and performed differential expression analyses using an ANOVA with multiple hypothesis testing correction. As predicted, the PCA found that the urinary proteome was clearly distinguishable compared to the serum and plasma proteomes, whereas serum and plasma were not differentiable (Supplementary Fig. S4). The proteomes of serum and plasma are potentially more interchangeable than urine, yet some proteins are unique to each biofluid. Despite the difference in global proteomes, there is a definite protein overlap between all three biofluids. This indicates that while the global proteomes are not interchangeable, individual proteins may be measured in multiple biofluids.

**Fig. 4:**
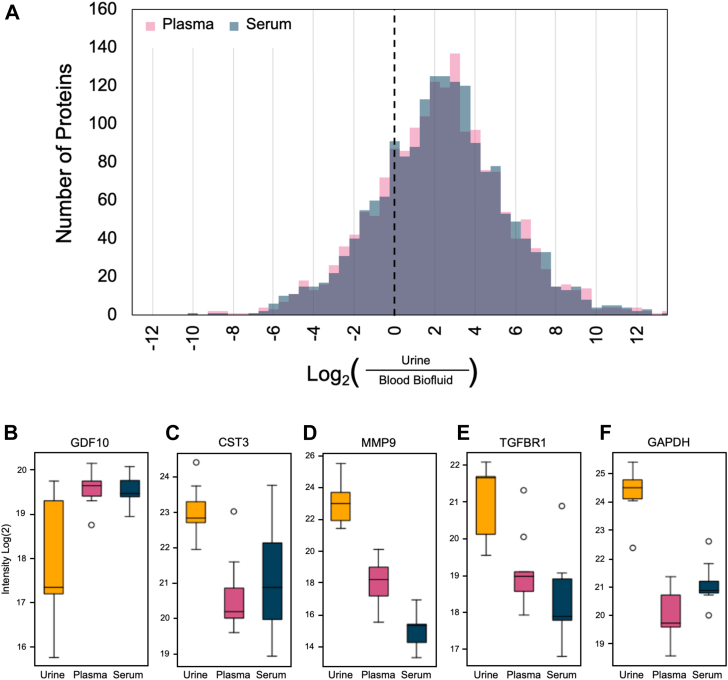
**Evaluation of protein overlap and relative intensities between biofluids and protein interchangeability.** (*A*) Histogram showing the log_2_ fold-change between urine and serum (*blue*) or plasma (*pink*). Boxplots show the relative intensity log_2_ of (*B*) growth differentiating factor 10, (*C*) cystatin C, (*D*) matrix metalloproteinase-9, (*E*) transforming growth factor B receptor 1, and (*F*) GAPDH across biofluids.

While the biofluids contain many of the same proteins, they are present at different relative intensities. Comparing the relative abundances of proteins across biofluids indicates which biofluids give the best signal for a designated protein of interest. This is important in streamlining candidate biomarker characterization, but is not always considered: instead, follow up is usually performed in the biofluid where the initial discovery was made. To understand how the overall relative intensities compared between urine and blood biofluids, we compared the fold-change differences for each protein between urine and plasma, or urine and serum (Fig. 4*A*). More proteins in urine had a higher relative intensity compared to serum or plasma, highlighting that urine may be an easier biofluid target compared to blood for certain protein measurements. As examples, growth differentiating factor 10, cystatin C, matrix metalloproteinase-9, transforming growth factor B receptor 1, and glyceraldehyde-3-phosphate dehydrogenase (GAPDH) were individually evaluated across biofluids (Fig. 4, *B*–*F*). For example, the relative intensities of growth differentiating factor 10 were highest in plasma, second highest in serum, and lower in urine (Fig. 4*B*). Conversely, the relative intensity of transforming growth factor B receptor 1 was highest ranking in urine, then plasma, then serum (Fig. 4*E*). This demonstrates that, depending on the protein of interest, certain biofluids will be more appropriate compared to other biofluids.

### Development of a Biofluid Database (CATalog)

Having determined that these three biofluids could be meaningfully compared, we sought to develop a database summarizing the measured intensities of proteins detected in urine, plasma, and serum. This database, named CATalog, is a bioinformatic tool that can be used to search for proteins of interest to aid in determining which biofluid to pursue based on the signal measured from the healthy cohort in this study. The objective of this database is to offer a way to interact with preliminary biofluid data in a digestible and efficient manner so that biomarker candidates can be selected for follow-up characterization. Moreover, this database is designed to be useful in assisting researchers in their decision of which biofluid to pursue regarding a specific protein of interest.

**Fig. 5:**
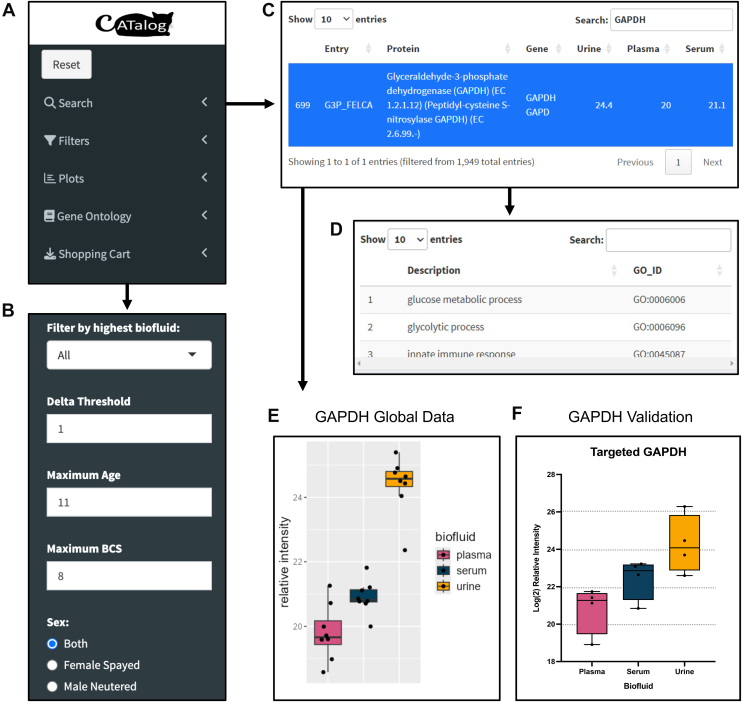
**CATalog demonstration of GAPDH.** Filter proteins by gene ontology (*A*). Add additional filters such as biofluid, age, body condition score, and sex (*B*). Search and explore proteins by name, ID, gene, or pathway (*C*). After selecting a protein, pathway information will appear (*D*) along with a visualization of the relative intensities in urine, plasma, and serum (*E*). Utilizing this database, a follow-up experiment demonstrates similar relative intensities of GAPDH in urine, serum, and plasma as described in the database (*F*).

The database contains 1949 proteins with Uniprot entry name, common protein name, gene name, and the average relative abundance measured in urine, plasma, and serum (Fig. 5). Based on these three averaged values, the database will indicate which biofluid(s) have the highest abundance displayed as an interactive boxplot. The database is interactive in that proteins can be searched, filtered, and scrolled through. To maximize information on each protein, a summary of additional protein information supplied by UniProt is displayed upon selecting a protein (Fig. 5, *C*–*E*). This application acts as a tool to inform researchers which biomarker candidates can be found in which biofluid, and which biofluid is optimal to pursue as a target. Since this database is derived from this study, this protocol offers a way to reproducibly process samples to detect any given protein. To demonstrate the utility of this interface, we performed a targeted experiment utilizing CATalog’s preliminary data.

To test CATalog, we targeted 10 proteins in a PRM experiment (Supplementary Table S2), including GAPDH, a common Western blot standard (61). CATalog’s output showed that urine had the highest relative intensity, with serum at the second highest relative intensity, and plasma had the third highest relative intensity (Fig. 5*E*). To evaluate if this trend would also be seen in a secondary targeted PRM experiment, all three biofluids were run with a smaller subset of cats targeting GAPDH. The results of the PRM assay found the same trend, where urine had the highest relative intensity, serum had the second highest relative intensity and plasma had the lowest relative intensity (Fig. 5*F*).

### Application of CATalog: Informed Biofluid Selection for Biomarker Discovery

We tested two potential biomarkers identified in the literature for CKD to demonstrate the utility of CATalog in selecting appropriate biofluids for biomarker discovery. CKD is a well-documented and highly prevalent disease in cats, affecting 30 to 80% of the feline population (61, 62). Here, we compare the relative abundance of retinol binding protein-4 (RBP4) and leptin across biofluids selected by CATalog in diseased and healthy cats.

**Fig. 6:**
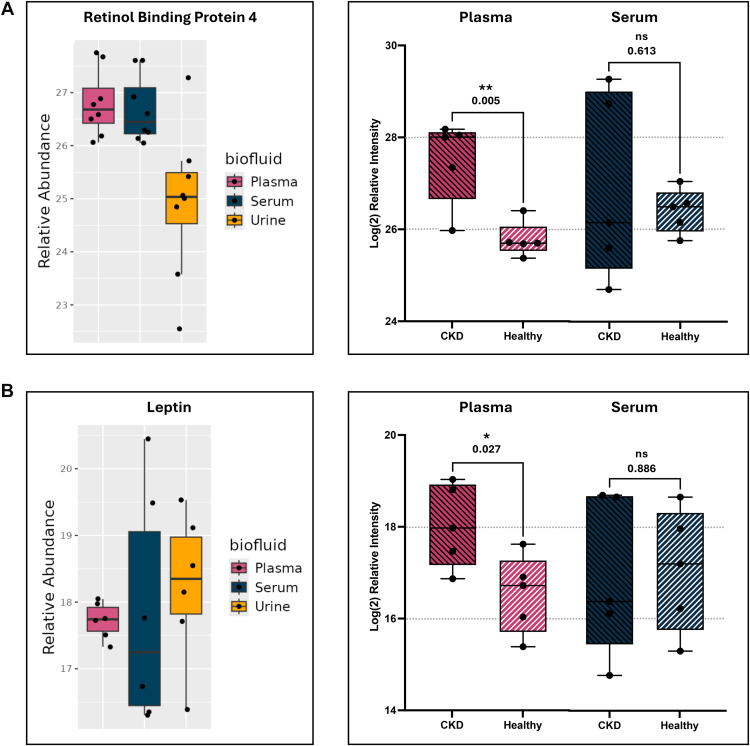
**Demonstration of the utility of CATalog for preliminary biofluid selection.** (*A*) Retinol binding protein 4 as shown in CATalog (*left*) and in a secondary targeted experiment (*right*) in chronic kidney disease and healthy cats in plasma and serum (plasma *p*-value = 0.005, serum *p*-value = 0.613). (*B*) Leptin as shown in CATalog (*left*) and in a secondary targeted experiment (*right*) in chronic kidney disease and healthy cats in plasma and serum (plasma *p*-value = 0.027, serum *p*-value = 0.886).

Retinol binding protein 4 (RBP4) is associated with obesity, insulin resistance, and renal dysfunction (63). Measuring RBP4 is relatively well documented in plasma and serum (64, 65, 66), so we would expect to see the highest relative intensities in blood biofluids. While RBP4 is commonly measured in humans as a biomarker for kidney function, when we search RBP4 in CATalog, the database shows elevated relative intensities in serum and plasma compared to urine (Fig. 6*A*). Focusing on blood, we performed a targeted PRM assay including five CKD cats and five healthy cats, showing that RBP4 was statistically differentiable between diseased and healthy cats in plasma (*p* = 0.005), but not in serum (*p* = 0.613) (Fig. 6*A*), and confirmed to be relatively low in urine (Supplementary Data 1). This demonstrates how CATalog may be able to characterize trends observed in the literature, but that disease biology still plays a role in which biofluid shows significant trends.

Cats with CKD commonly suffer from inappetence, which may be a result of an imbalance of orexigenic and anorexigenic hormones (67). Leptin, a hunger suppressing hormone, is a key in regulating appetite (68). Because leptin is renally excreted (69), damaged kidney function can cause accumulation of leptin in the bloodstream (70). While it is well documented that leptin should be measured in blood biofluids, there is not a clear consensus on if plasma or serum (71, 72, 73). In this case, CATalog offers some clarity on whether to choose plasma or serum. The leptin data in CATalog shows that plasma has much lower variability between bioreplicates compared to serum, indicating that plasma may have more statistical power compared to serum (Fig. 6*B*). In the PRM assay, while both blood biofluids were successfully detected leptin, plasma showed a significant difference between CKD and healthy cats (*p*-value = 0.027) compared to serum (*p*-value = 0.886). In this case, CATalog was able to predict which biofluid would yield the highest statistical power based on the observed variability.

### Study Limitations

While currently configured for interpreting proteins in feline biofluids, we are actively applying these methodologies to a human cohort to expand the variety of data available in CATalog. The data in CATalog is meant to represent a baseline demonstration of what normal relative intensities look like in the different biofluids of healthy cats. However, a larger number of cats is needed to represent the total cat population, including cats of varying ages, sexes (including intact and neutered), and disease states. Additionally, the inclusion of obese cats will be vital in representing the feline population, since over half of the feline population suffers from obesity (74). It should also be noted that while choosing a biofluid based on the highest relative intensity can be informative, other factors such as disease involvement and ease of obtainability should also be considered to ensure that biofluid selection produces meaningful biological conclusions.

## Conclusions

This study provides a mass spectrometry-based workflow for analyzing biofluids in parallel to evaluate biofluid surrogacy for any given protein assay target. This harmonization of urine and blood biofluids opens avenues for applications in biofluids such as cerebrospinal fluid, saliva, and tears. Interactive databases allow for large quantities of data, such as global mass spectrometry data, to be summarized and easily searched. While many databases have been released, to our knowledge, there are none that directly compare the relative quantification of proteins between biofluids. CATalog offers a way to communicate preliminary data on biomarker candidates, allowing researchers to implement and validate clinical assays more efficiently. While currently demonstrated using a dataset based on healthy cats, in the future this tool can be extended to other clinically important species, including humans. Using this workflow, our data shows how biofluid selection can impact protein measurement quality, which can be assessed before assay development. We highlight that in some cases, urine represents blood biofluids without requiring venipuncture or sample depletion of high abundant proteins. Given its consistently higher relative protein abundances, we emphasize the potential of urine as a “goldmine” of biomarker candidates. Considering the substantial overlap of urinary proteins with blood proteins, we encourage the consideration of urine as a less invasive, predepleted biofluid.

## Institutional Review Board Statement

The use of all cats in this study was approved by The Ohio State University International Animal Care and Use Committee (International Animal Care and Use Committee #2020A00000042).

## Data Availability

All raw data is publicly available on mass spectrometry interactive virtual environment using dataset identifier MSV000096055 (reviewer password: “CATalog”) and at ProteomeXchange using the dataset identifier PXD056668. CATalog is freely available at https://searlelab.shinyapps.io/CATalog/. Open-source software developed for this project is publicly available as part of the EncyclopeDIA project at https://bitbucket.org/searleb/encyclopedia and as the CATalog project at https://github.com/searlelab/CATalog.

## Supplemental Data

This article contains supplemental data.

## Conflict of Interest

The authors declare no competing interests.
