# Supplementary material for: Harmonized Peptide Libraries Enable Practical Biofluid Selection for Developing Biomarker Assays
Source: Mol Cell Proteomics. 2025 Oct 12;24(12):101086. doi: 10.1016/j.mcpro.2025.101086 (PMC12702218; doi:10.1016/j.mcpro.2025.101086)
Supplement: Supplementary Figures and Tables [file mmc2.pdf]

Supplementary Data for:

Harmonized peptide libraries enable practical biofluid selection for developing biomarker assays

Katelyn B. Brusach,[1,†] Ariana E. Shannon,[2,3,†] Alex W. Joyce,[2,3] Jessica M. Quimby,[1] Brian C. Searle[2,3,†,*]

[1]Department of Veterinary Clinical Sciences, The Ohio State University College of Veterinary Medicine, Columbus, OH, 43210, USA.

[2]Pelotonia Institute for Immuno-Oncology, The Ohio State University Comprehensive Cancer Center, Columbus, OH, 43210, USA.

[3]Department of Biomedical Informatics, The Ohio State University Medical Center, Columbus, OH, 43210, USA.

[*]Corresponding Address: Brian C. Searle, Ph.D., searle.brian@mayo.edu

†Current address: Department of Quantitative Health Sciences, Mayo Clinic, Rochester, MN 55905, USA.

Supplementary Table 1: Cat health information for global (**A**) and targeted (**B**) datasets. MN = Male Neutered, FS = Female Spayed, USG = Urine Specific Gravity, CKD = Chronic Kidney Disease. Age is represented in years. Normal ranges for USG are >1.035 and <1.6 mg/dl for serum creatinine.

A

Cat	Health Status	Age	Sex	USG	Serum Creatinine
B	Healthy	7	FS	1.062	1
C	Healthy	5	MN	1.06	1.5
D	Healthy	3	MN	1.046	1.5
H	Healthy	11	MN	1.06	1.2
I	Healthy	8	FS	1.063	1.2
M	Healthy	1	MN	1.069	0.9
N	Healthy	10	FS	1.045	1.3
O	Healthy	3	FS	1.045	1.5

B

Cat	Health Status	Age	Sex	USG	Serum Creatinine
A	CKD	13	MN	1.043	1.6
B	Healthy	7	FS	1.062	1
C	Healthy	5	MN	1.06	1.5
E	Healthy	3	MN	1.039	1.6
F	CKD	6	MN	1.041	2
G	CKD	20	FS	1.012	3.1
I	Healthy	8	FS	1.063	1.2
J	CKD	8	MN	1.01	4
O	Healthy	3	FS	1.045	1.5
P	CKD	13	MN	1.04	1.7

Supplementary Table 2: Metadata for ten proteins of interest targeted in PRM experiment, including chronic kidney disease and healthy cats. Included (from left to right) are the Uniprot Entry name, Entry ID, Protein Name, Gene Name, Uniprot Review Status, and Protein Length.

Uniprot Entry Name	Entry	Protein names	Gene Names	Reviewed	Length
RET4_FELCA	M5AXY1	Retinol binding protein 4 (RBP4)	RBP4	reviewed	201
SAA_FELCA	P19707	Serum amyloid A protein (SAA)	SAA1	reviewed	129
G3P_FELCA	Q9N2D5	Glyceraldehyde-3-phosphate dehydrogenase (GAPDH)	GAPDH	reviewed	333
DPP4_FELCA	Q9N2I7	Dipeptidyl peptidase 4 (DPP IV)	DPP4	reviewed	765
A0A2I2UP60_FELCA	A0A2I2UP60	Na(+)/H(+) exchange regulatory cofactor (NHERF1)	NHERF1	unreviewed	366
A0A5F5Y5F5_FELCA	A0A5F5Y5F5	Matrix metalloproteinase-9	MMP9	unreviewed	660
D5MTH1_FELCA	D5MTH1	Cystatin C	CST3	unreviewed	147
M3W230_FELCA	M3W230	Uromodulin	UMOD	unreviewed	642
M3W2Z1_FELCA	M3W2Z1	Vitamin D-binding protein	GC	unreviewed	474
LEP_FELCA	Q9N2C1	Leptin	LEP	reviewed	167

Supplementary Table 3: Peptide target list for the ten proteins in the targeted PRM experiment.

Compound	m/z	z	RT Time (min)	Window (min)	HCD Collision Energies (%)	Maximum Injection Time (ms)
DPNGLPPDVQK	590.8039	2	34.135	10	30	120
YWGVASFLQK	600.3164	2	88.042	10	30	120
FSGTWYAMAK	581.7735	2	64.479	10	30	240
GNDDHWIIDTDYDTYAVQYSC[+57.021464]R	903.5504	3	87.123	10	30	120
LIVHNGYC[+57.021464]DGKSEQNIL	654.4913	3	53.928	10	30	120
GPGGAWAAK	408.2139	2	24.56	10	30	120
EANYIGADK	491.2378	2	23.765	10	30	240
EANYIGADKYFHAR	552.7706	3	45.116	10	30	120
VISDARENSQR	426.0531	3	17.164	10	30	120
RGPGGAWAAK	486.2645	2	19.619	10	30	120
VAGLDFIPGLHPVLSLSK	622.1961	3	96.56	20	30	240
GAAQNIIPASTGAAK	685.8753	2	37.251	10	30	120
VGVNGFGR	403.7194	2	32.709	10	30	240
LVINGKPITIFQER	543.8225	3	69.969	10	30	120
VIPELNGK	435.7582	2	35.213	10	30	120
LISWYDNEFGYSNR	882.9048	2	86.132	10	30	120
WEYYDSVYTER	756.3279	2	68.686	10	30	120
LGTFEVEDQIEAAR	789.8939	2	70.809	10	30	240
VLEDNSALDK	552.7826	2	28.763	10	30	120
TYTLTDYLK	559.7924	2	71.828	10	30	120
IISNEDGYK	520.2587	2	24.332	10	30	120
LLVVDPETDER	643.8353	2	52.232	10	30	240
AVDPDSPAEASGLR	693.3388	2	38.618	10	30	120
LVEVNGENVEK	615.8222	2	31.641	10	30	120
KNELFSNF	500.2507	2	64.5	10	30	120
IVEVNGVC[+57.021464]MEGK	668.3258	2	45.165	10	30	120
LYGFC[+57.021464]PTR	507.7473	2	49.495	10	30	240
TLC[+57.021464]SFQIYTVPWMGK	916.4495	2	96.825	10	30	120
KTLC[+57.021464]SFQIYTVPWMGK	654.1671	3	89.93	10	30	240
SQPNLDTC[+57.021464]PFHDQPHLMR	732.1703	3	54.432	10	30	120
GDRDWISVVTPAR	491.7599	3	64.426	10	30	240
DWISVVTPAR	572.8115	2	77.956	10	30	120
DSTIQVVENGESPQGR	573.1113	3	45.818	10	30	120
FVGQGGVR	410.7272	2	22.628	10	30	120
TKDSTIQVVENGESPQGR	649.4922	3	33.246	10	30	120
AIDQYIFELSR	678.3537	2	88.661	10	30	120
ELSSFIDK	470.2451	2	48.591	10	30	120
ILEPTLK	407.7577	2	38.88	10	30	120
TPLPEVFLSK	566.3264	2	73.668	10	30	120
HQPQEFPTYVEPTNDEIC[+57.021464]EAFR	903.5746	3	80.809	10	30	240

Supplementary Table 4: The number of proteins detected between three methods of sample preparation (Acetone, Perchloric Acid, Acetone+Perchloric Acid) in urine, serum, and plasma.

Treatment	Urine	Plasma	Serum
Acetone	1361	526	561
Acetone + Perchloric Acid	1024	509	575
Perchloric Acid	825	543	538

Supplementary Figure 1: Protein overlap between three types of sample preparation (Acetone, Perchloric Acid, Acetone+Perchloric Acid) in urine, plasma, and serum.

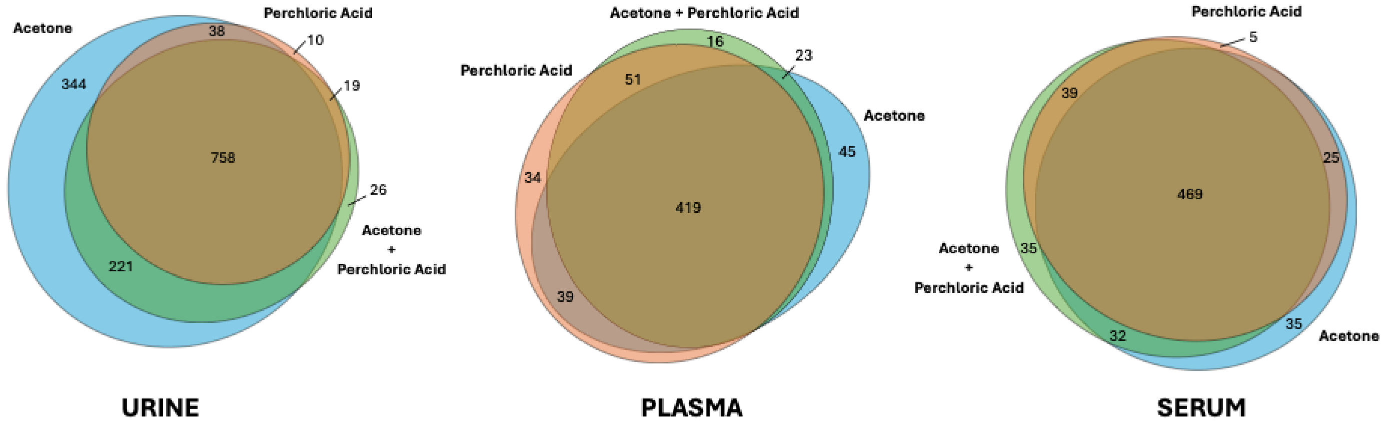

Supplementary Figure 2: Retention time differences (y-axis, minutes) between urine, plasma, and serum standard samples.

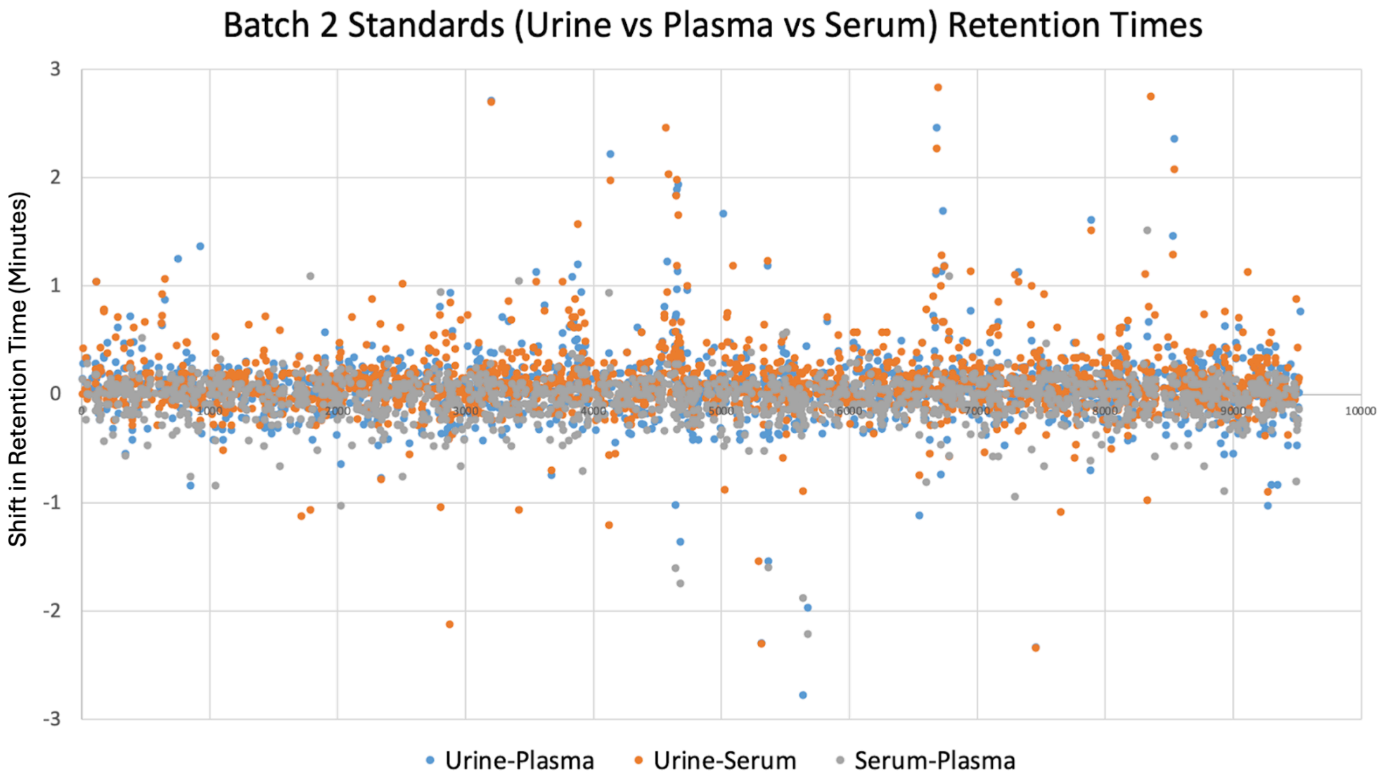

Supplementary Figure 3: Heatmap evaluating confounding variables. From top to bottom, age, biofluid, body condition score, cat bioreplicates (sample ID letter), and sex.

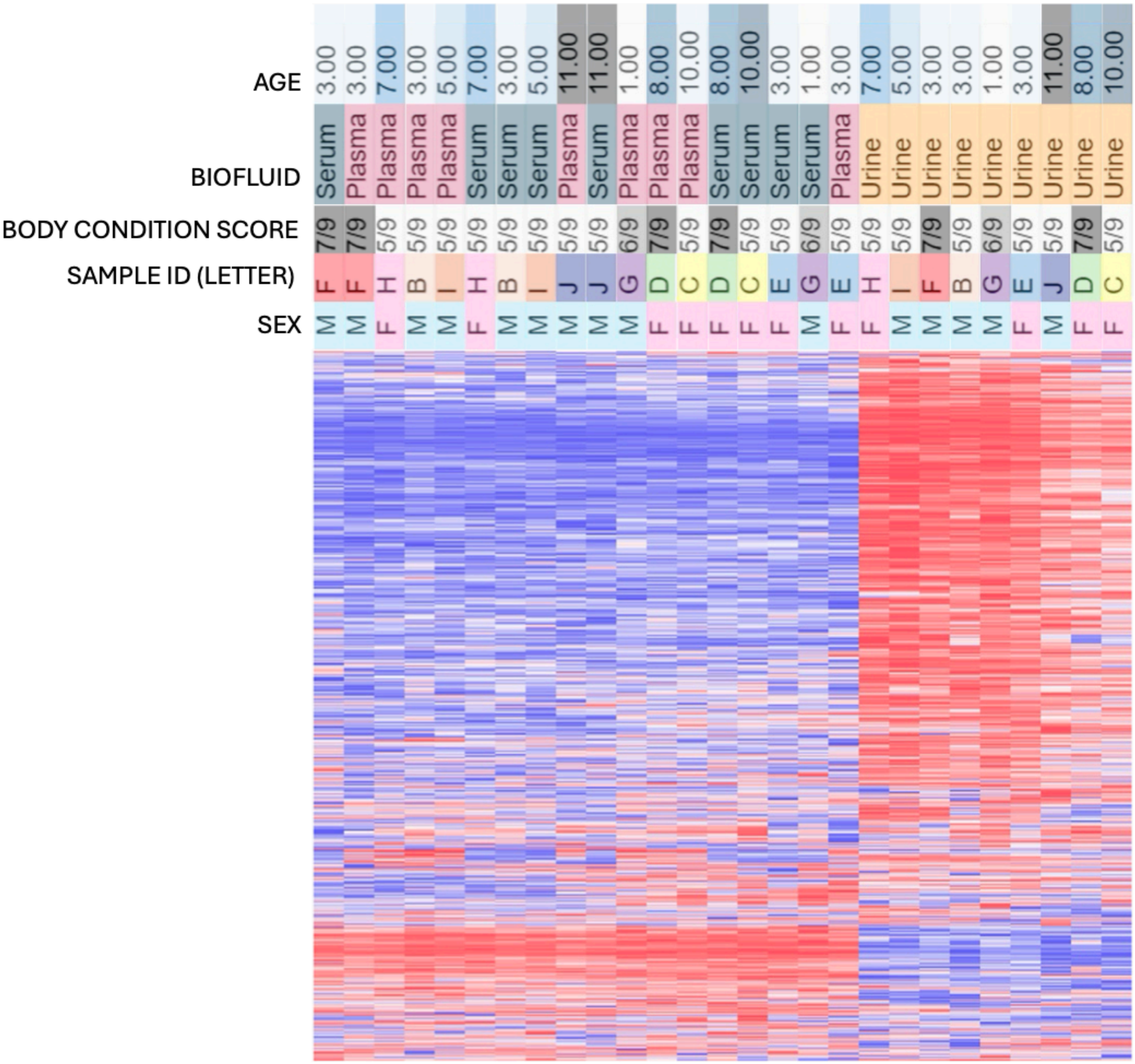

Supplementary Figure 4: PCA analysis between cat bioreplicates of urine, plasma, and serum.

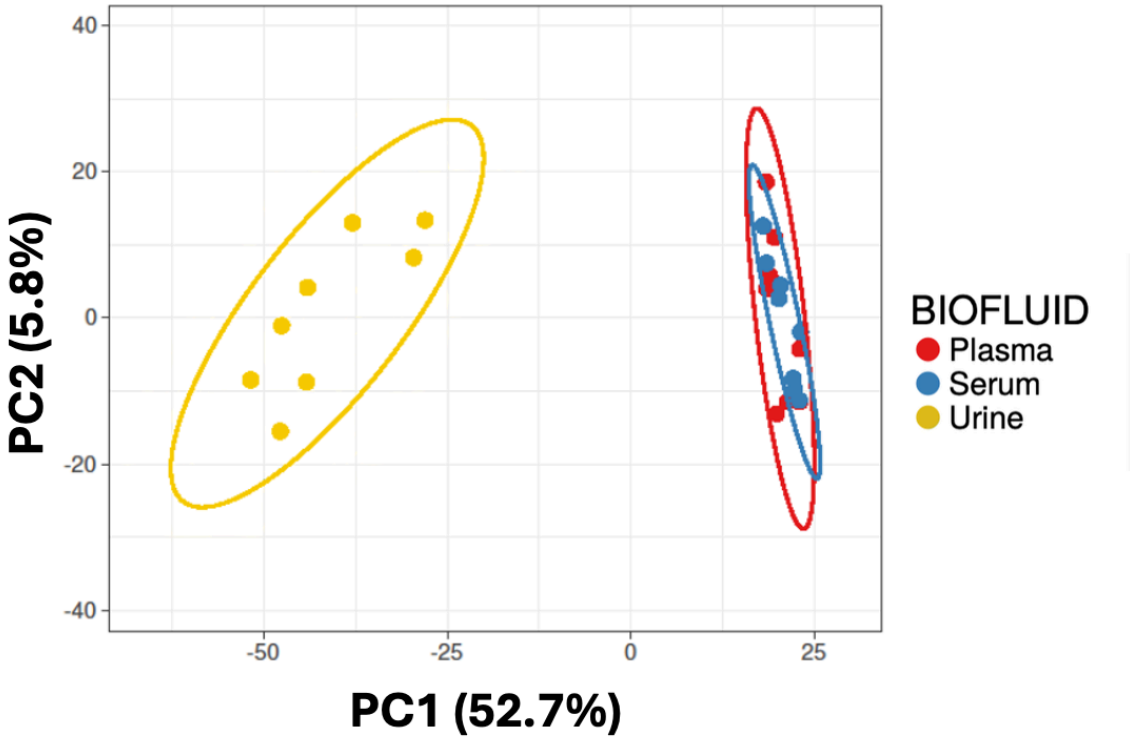

Supplementary Figure 5: Annotated chromatograms to support the identification of single unique peptides.

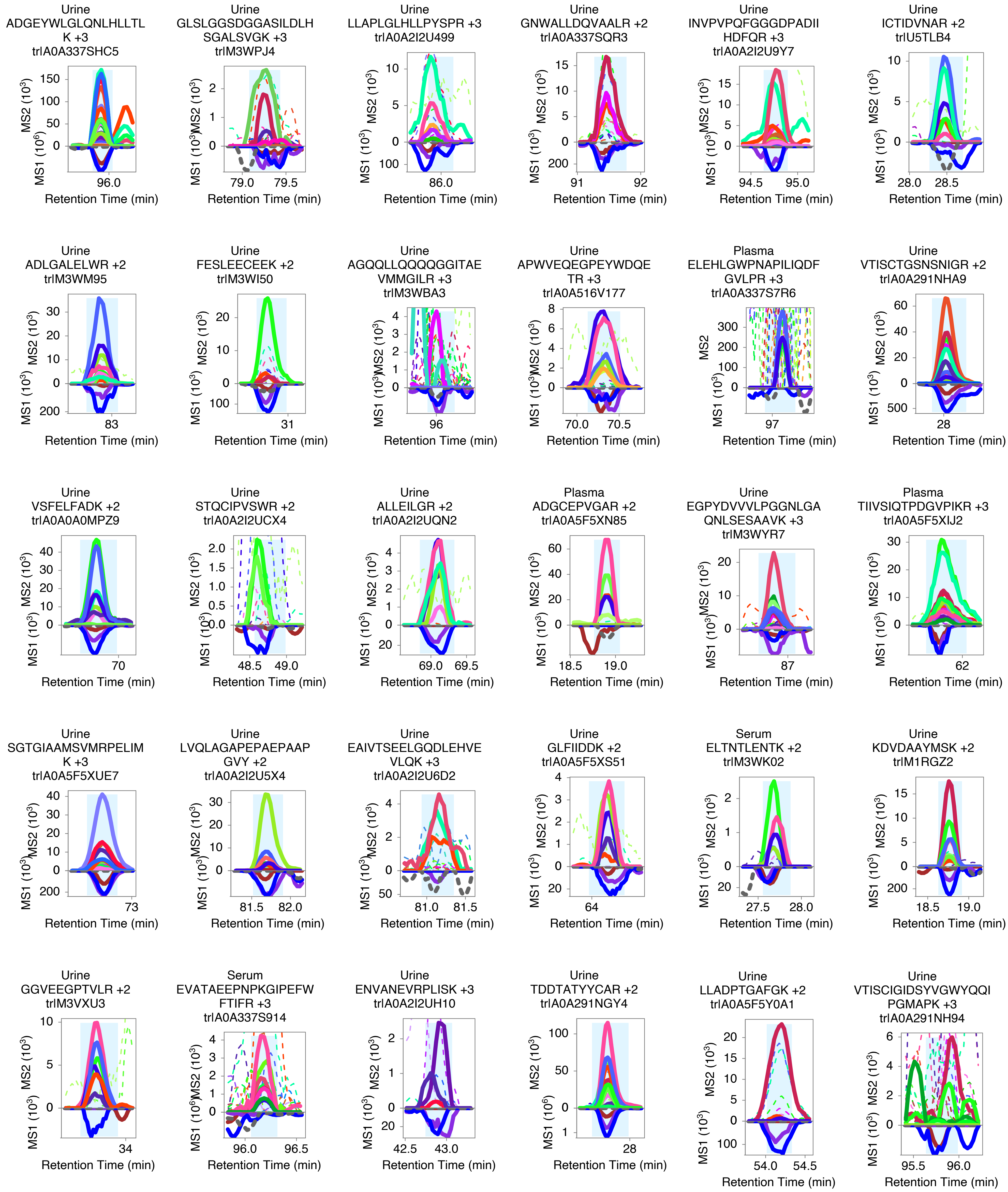

Urine
ADGEYWLGLQNLHLLTL
K +3
trlA0A337SHC5
MS2 (10³)
MS1 (10⁶)
150
100
50
0
96.0
Retention Time (min)
Urine
GLSLGGSDGGASILDLH
SGALSVGK +3
trlM3WPJ4
MS1 (10³)MS2 (10³)
2
1
0
79.0
79.5
Retention Time (min)
Urine
LLAPLGLHLLPYSPR +3
trlA0A2I2U499
MS1 (10³) MS2 (10³)
10
5
0
100
86.0
Retention Time (min)
Urine
GNWALLDQVAALR +2
trlA0A337SQR3
MS1 (10³) MS2 (10³)
15
10
5
0
200
91
92
Retention Time (min)
Urine
INVPVPQFGGGDPADII
HDFQR +3
trlA0A2I2U9Y7
MS1 (10³)MS2 (10³)
15
10
5
0
94.5
95.0
Retention Time (min)
Urine
ICTIDVNAR +2
trlU5TLB4
MS1 (10³) MS2 (10³)
10
5
0
28.0
28.5
Retention Time (min)
Urine
ADLGALELWR +2
trlM3WM95
MS1 (10³) MS2 (10³)
30
20
10
0
200
83
Retention Time (min)
Urine
FESLEECEEK +2
trlM3WI50
MS1 (10³) MS2 (10³)
20
10
0
100
31
Retention Time (min)
Urine
AGQQLLQQQQGGITAE
VMMGILR +3
trlM3WBA3
MS1 (10³)MS2 (10³)
4
3
2
1
0
96
Retention Time (min)
Urine
APWVEQEGPEYWDQE
TR +3
trlA0A516V177
MS1 (10³)MS2 (10³)
8
6
4
2
0
70.0
70.5
Retention Time (min)
Plasma
ELEHLGWPNAPILIQDF
GVLPR +3
trlA0A337S7R6
MS2
MS1 (10³)
300
200
100
0
97
Retention Time (min)
Urine
VTISCTGSNSNIGR +2
trlA0A291NHA9
MS1 (10³) MS2 (10³)
60
40
20
0
500
28
Retention Time (min)
Urine
VSFELFADK +2
trlA0A0A0MPZ9
MS1 (10³) MS2 (10³)
40
30
20
10
0
70
Retention Time (min)
Urine
STQCIPVSWR +2
trlA0A2I2UCX4
MS1 (10³) MS2 (10³)
2.0
1.5
1.0
0.5
0.0
48.5
49.0
Retention Time (min)
Urine
ALLEILGR +2
trlA0A2I2UQN2
MS1 (10³) MS2 (10³)
4
3
2
1
0
20
69.0
69.5
Retention Time (min)
Plasma
ADGCEPVGAR +2
trlA0A5F5XN85
MS1 (10³) MS2 (10³)
60
40
20
0
18.5
19.0
Retention Time (min)
Urine
EGPYDVVVLPGGNLGA
QNLSESAAVK +3
trlM3WYR7
MS1 (10³)MS2 (10³)
20
10
0
87
Retention Time (min)
Plasma
TIIVSIQTPDGVPIKR +3
trlA0A5F5XIJ2
MS1 (10³) MS2 (10³)
30
20
10
0
62
Retention Time (min)
Urine
SGTGIAAMSVMRPELIM
K +3
trlA0A5F5XUE7
MS1 (10³)MS2 (10³)
40
30
20
10
0
200
73
Retention Time (min)
Urine
LVQLAGAPEPAEPAAP
GVY +2
trlA0A2I2U5X4
MS1 (10³)MS2 (10³)
30
20
10
0
81.5
82.0
Retention Time (min)
Urine
EAIVTSEELGQDLEHVE
VLQK +3
trlA0A2I2U6D2
MS1 (10³)MS2 (10³)
4
2
0
50
81.0
81.5
Retention Time (min)
Urine
GLFIIDDK +2
trlA0A5F5XS51
MS1 (10³) MS2 (10³)
4
3
2
1
0
20
64
Retention Time (min)
Serum
ELTNTLENTK +2
trlM3WK02
MS1 (10³) MS2 (10³)
2
1
0
20
27.5
28.0
Retention Time (min)
Urine
KDVDAAYMSK +2
trlM1RGZ2
MS1 (10³) MS2 (10³)
15
10
5
0
200
18.5
19.0
Retention Time (min)
Urine
GGVEEGPTVLR +2
trlM3VXU3
MS1 (10³) MS2 (10³)
10
5
0
34
Retention Time (min)
Serum
EVATAEEPNPKGIPEFW
FTIFR +3
trlA0A337S914
MS1 (10⁶)MS2 (10³)
4
3
2
1
0
96.0
96.5
Retention Time (min)
Urine
ENVANEVRPLISK +3
trlA0A2I2UH10
MS1 (10³) MS2 (10³)
2
1
0
20
42.5
43.0
Retention Time (min)
Urine
TDDTATYYCAR +2
trlA0A291NGY4
MS1 (10⁶) MS2 (10³)
100
50
0
1
28
Retention Time (min)
Urine
LLADPTGAFGK +2
trlA0A5F5Y0A1
MS1 (10³) MS2 (10³)
20
15
10
5
0
100
54.0
54.5
Retention Time (min)
Urine
VTISCIGIDSYVGWYQQI
PGMAPK +3
trlA0A291NH94
MS1 (10³) MS2 (10³)
6
4
2
0
95.5
96.0
Retention Time (min)

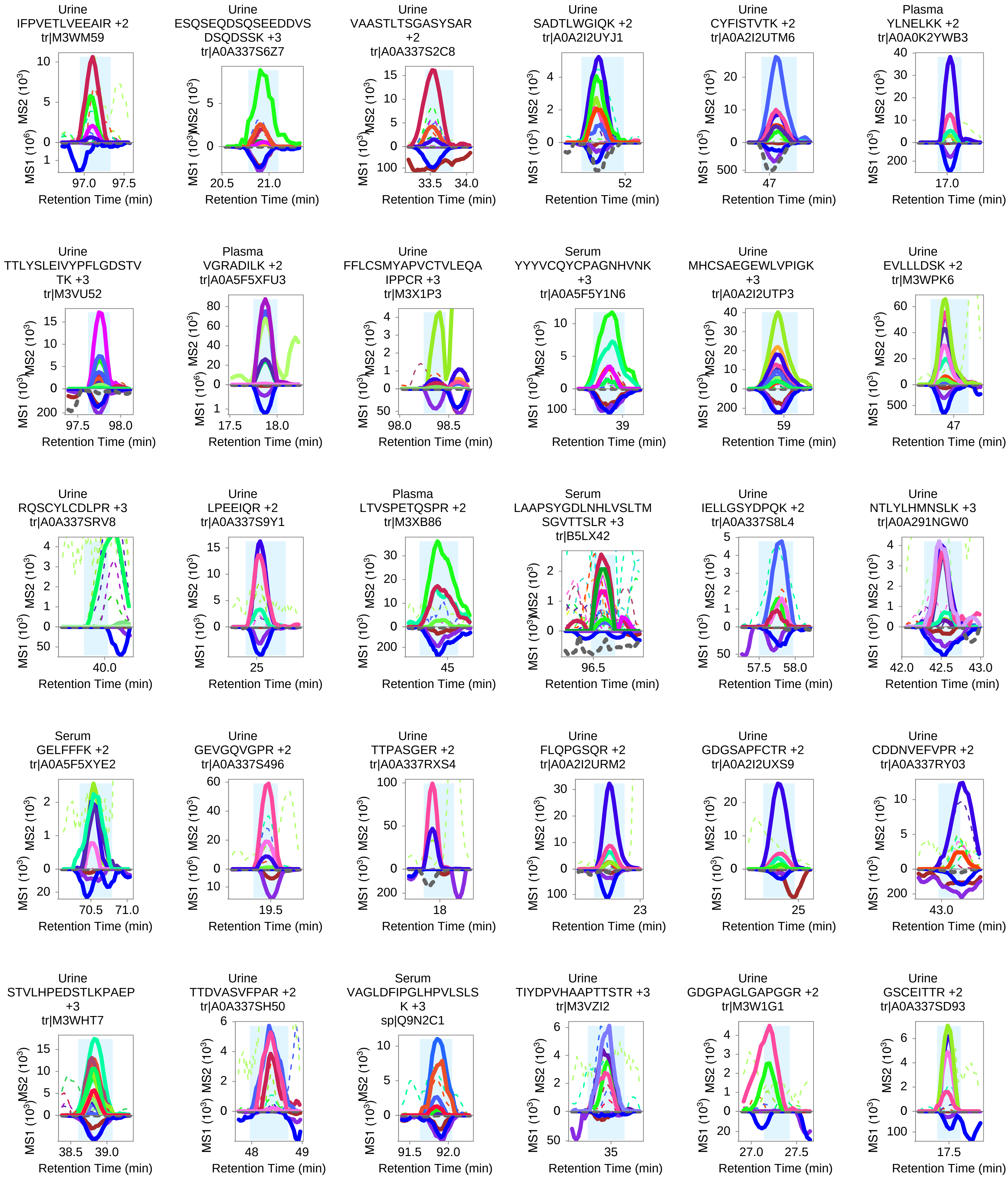

Urine
IFPVETLVEEAIR +2
tr|M3WM59
MS2 (10³)
MS1 (10⁶)
Retention Time (min)
Urine
ESQSEQDSQSEEDDVS
DSQDSSK +3
tr|A0A337S6Z7
MS2 (10³)
MS1 (10³)
Retention Time (min)
Urine
VAASTLTSGASYSAR
+2
tr|A0A337S2C8
MS2 (10³)
MS1 (10³)
Retention Time (min)
Urine
SADTLWGIQK +2
tr|A0A2I2UYJ1
MS2 (10³)
MS1 (10³)
Retention Time (min)
Urine
CYFISTVTK +2
tr|A0A2I2UTM6
MS2 (10³)
MS1 (10³)
Retention Time (min)
Plasma
YLNELKK +2
tr|A0A0K2YWB3
MS2 (10³)
MS1 (10³)
Retention Time (min)
Urine
TTLYSLEIVYPFLGDSTV
TK +3
tr|M3VU52
MS2 (10³)
MS1 (10³)
Retention Time (min)
Plasma
VGRADILK +2
tr|A0A5F5XFU3
MS2 (10³)
MS1 (10⁶)
Retention Time (min)
Urine
FFLCSMYAPVCTVLEQA
IPPCR +3
tr|M3X1P3
MS2 (10³)
MS1 (10³)
Retention Time (min)
Serum
YYYVCQYCPAGNHVNK
+3
tr|A0A5F5Y1N6
MS2 (10³)
MS1 (10³)
Retention Time (min)
Urine
MHCSAEGEWLVPIGK
+3
tr|A0A2I2UTP3
MS2 (10³)
MS1 (10³)
Retention Time (min)
Urine
EVLLLDSK +2
tr|M3WPK6
MS2 (10³)
MS1 (10³)
Retention Time (min)
Urine
RQSCYLCDLPR +3
tr|A0A337SRV8
MS2 (10³)
MS1 (10³)
Retention Time (min)
Urine
LPEEIQR +2
tr|A0A337S9Y1
MS2 (10³)
MS1 (10³)
Retention Time (min)
Plasma
LTVSPETQSPR +2
tr|M3XB86
MS2 (10³)
MS1 (10³)
Retention Time (min)
Serum
LAAPSYGDLNHLVSLTM
SGVTTSLR +3
tr|B5LX42
MS2 (10³)
MS1 (10³)
Retention Time (min)
Urine
IELLGSYDPQK +2
tr|A0A337S8L4
MS2 (10³)
MS1 (10³)
Retention Time (min)
Urine
NTLYLHMNSLK +3
tr|A0A291NGW0
MS2 (10³)
MS1 (10³)
Retention Time (min)
Serum
GELFFFK +2
tr|A0A5F5XYE2
MS2 (10³)
MS1 (10³)
Retention Time (min)
Urine
GEVGQVGPR +2
tr|A0A337S496
MS2 (10³)
MS1 (10⁶)
Retention Time (min)
Urine
TTPASGER +2
tr|A0A337RXS4
MS2 (10³)
MS1 (10³)
Retention Time (min)
Urine
FLQPGSQR +2
tr|A0A2I2URM2
MS2 (10³)
MS1 (10³)
Retention Time (min)
Urine
GDGSAPFCTR +2
tr|A0A2I2UXS9
MS2 (10³)
MS1 (10³)
Retention Time (min)
Urine
CDDNVEFVPR +2
tr|A0A337RY03
MS2 (10³)
MS1 (10³)
Retention Time (min)
Urine
STVLHPEDSTLKPAEP
+3
tr|M3WHT7
MS2 (10³)
MS1 (10³)
Retention Time (min)
Urine
TTDVASVFPAR +2
tr|A0A337SH50
MS2 (10³)
MS1 (10³)
Retention Time (min)
Serum
VAGLDFIPGLHPVLSLS
K +3
sp|Q9N2C1
MS2 (10³)
MS1 (10³)
Retention Time (min)
Urine
TIYDPVHAAPTTSTR +3
tr|M3VZI2
MS2 (10³)
MS1 (10³)
Retention Time (min)
Urine
GDGPAGLGAPGGR +2
tr|M3W1G1
MS2 (10³)
MS1 (10³)
Retention Time (min)
Urine
GSCEITTR +2
tr|A0A337SD93
MS2 (10³)
MS1 (10³)
Retention Time (min)

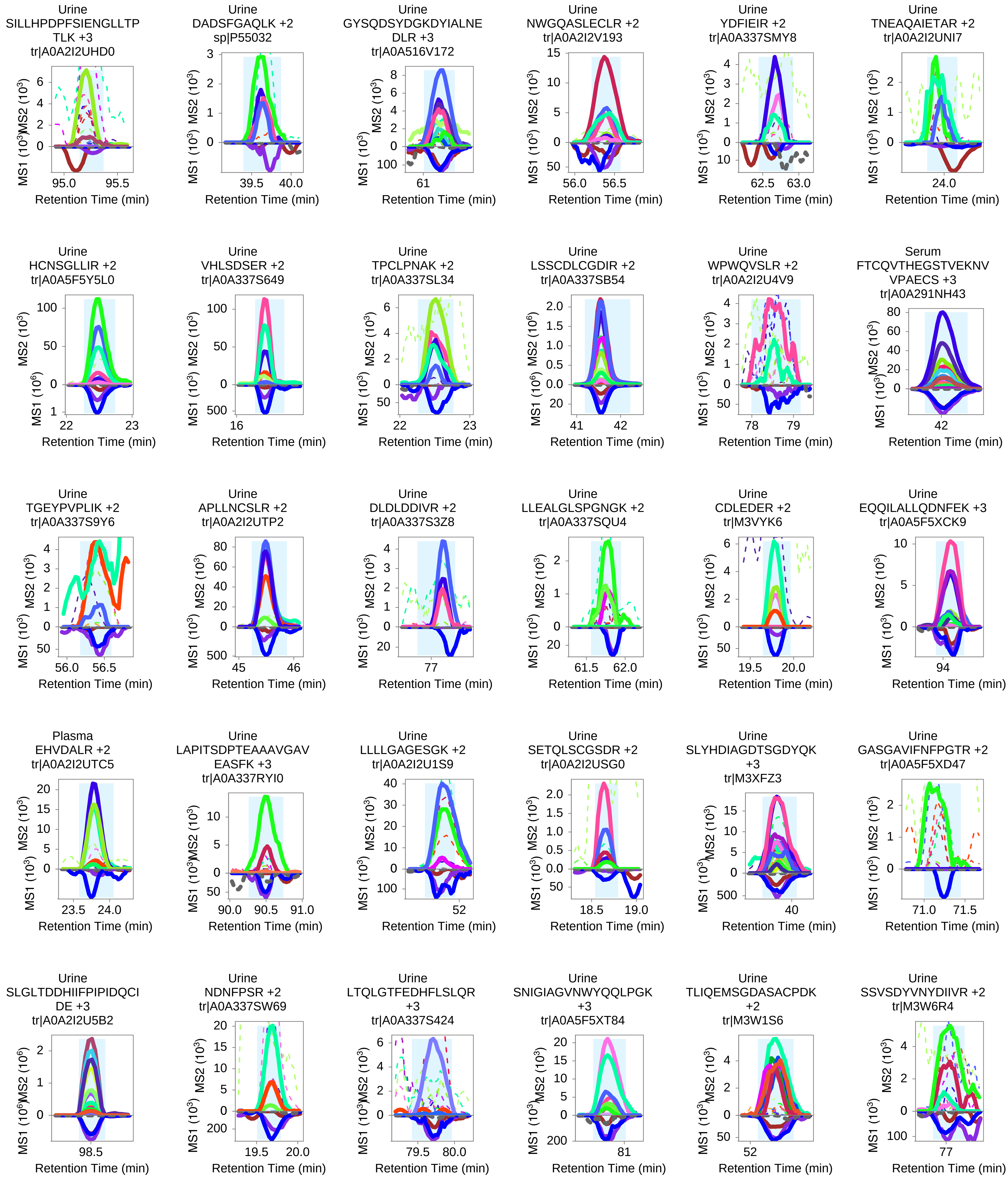
Urine
SILLHPDPFSIENGLLTP
TLK +3
tr|A0A2I2UHD0
MS1 (10³) MS2 (10³)
Retention Time (min)
Urine
DADSFGAQLK +2
sp|P55032
MS1 (10³) MS2 (10³)
Retention Time (min)
Urine
GYSQDSYDGKDYIALNE
DLR +3
tr|A0A516V172
MS1 (10³) MS2 (10³)
Retention Time (min)
Urine
NWGQASLECLR +2
tr|A0A2I2V193
MS1 (10³) MS2 (10³)
Retention Time (min)
Urine
YDFIEIR +2
tr|A0A337SMY8
MS1 (10³) MS2 (10³)
Retention Time (min)
Urine
TNEAQAIETAR +2
tr|A0A2I2UNI7
MS1 (10³) MS2 (10³)
Retention Time (min)
Urine
HCNSGLLIR +2
tr|A0A5F5Y5L0
MS1 (10⁶) MS2 (10³)
Retention Time (min)
Urine
VHLSDSER +2
tr|A0A337S649
MS1 (10³) MS2 (10³)
Retention Time (min)
Urine
TPCLPNAK +2
tr|A0A337SL34
MS1 (10³) MS2 (10³)
Retention Time (min)
Urine
LSSCDLCGDIR +2
tr|A0A337SB54
MS1 (10⁶) MS2 (10⁶)
Retention Time (min)
Urine
WPWQVSLR +2
tr|A0A2I2U4V9
MS1 (10³) MS2 (10³)
Retention Time (min)
Serum
FTCQVTHEGSTVEKNV
VPAECS +3
tr|A0A291NH43
MS1 (10³) MS2 (10³)
Retention Time (min)
Urine
TGEYPVPLIK +2
tr|A0A337S9Y6
MS1 (10³) MS2 (10³)
Retention Time (min)
Urine
APLLNCSLR +2
tr|A0A2I2UTP2
MS1 (10³) MS2 (10³)
Retention Time (min)
Urine
DLDLDDIVR +2
tr|A0A337S3Z8
MS1 (10³) MS2 (10³)
Retention Time (min)
Urine
LLEALGLSPGNGK +2
tr|A0A337SQU4
MS1 (10³) MS2 (10³)
Retention Time (min)
Urine
CDLEDER +2
tr|M3VYK6
MS1 (10³) MS2 (10³)
Retention Time (min)
Urine
EQQILALLQDNFEK +3
tr|A0A5F5XCK9
MS1 (10³) MS2 (10³)
Retention Time (min)
Plasma
EHVDALR +2
tr|A0A2I2UTC5
MS1 (10³) MS2 (10³)
Retention Time (min)
Urine
LAPITSDPTEAAAVGAV
EASFK +3
tr|A0A337RYI0
MS1 (10³) MS2 (10³)
Retention Time (min)
Urine
LLLLGAGESGK +2
tr|A0A2I2U1S9
MS1 (10³) MS2 (10³)
Retention Time (min)
Urine
SETQLSCGSDR +2
tr|A0A2I2USG0
MS1 (10³) MS2 (10³)
Retention Time (min)
Urine
SLYHDIAGDTSGDYQK
+3
tr|M3XFZ3
MS1 (10³) MS2 (10³)
Retention Time (min)
Urine
GASGAVIFNFPGTR +2
tr|A0A5F5XD47
MS1 (10³) MS2 (10³)
Retention Time (min)
Urine
SLGLTDDHIIFPIPIDQCI
DE +3
tr|A0A2I2U5B2
MS1 (10⁶) MS2 (10⁶)
Retention Time (min)
Urine
NDNFPSR +2
tr|A0A337SW69
MS1 (10³) MS2 (10³)
Retention Time (min)
Urine
LTQLGTFEDHFLSLQR
+3
tr|A0A337S424
MS1 (10³) MS2 (10³)
Retention Time (min)
Urine
SNIGIAGVNWYQQLPGK
+3
tr|A0A5F5XT84
MS1 (10³) MS2 (10³)
Retention Time (min)
Urine
TLIQEMSGDASACPDK
+2
tr|M3W1S6
MS1 (10³) MS2 (10³)
Retention Time (min)
Urine
SSVSDYVNYDIIVR +2
tr|M3W6R4
MS1 (10³) MS2 (10³)
Retention Time (min)

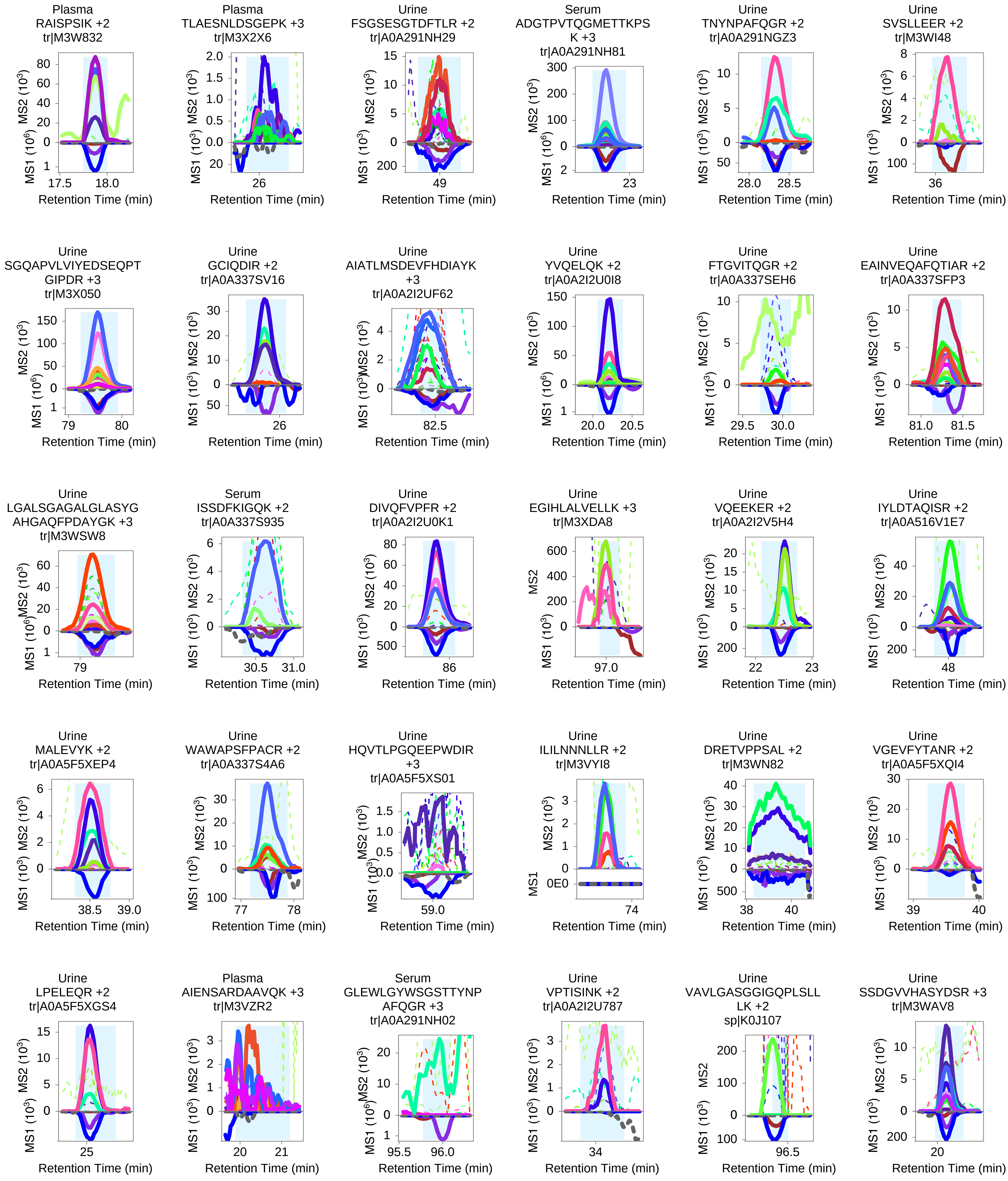
Plasma
RAISPSIK +2
tr|M3W832
Plasma
TLAESNLDSGEPK +3
tr|M3X2X6
Urine
FSGSESGTDFTLR +2
tr|A0A291NH29
Serum
ADGTPVTQGMETTKPS
K +3
tr|A0A291NH81
Urine
TNYNPAFQGR +2
tr|A0A291NGZ3
Urine
SVSLLEER +2
tr|M3WI48
Urine
SGQAPVLVIYEDSEQPT
GIPDR +3
tr|M3X050
Urine
GCIQDIR +2
tr|A0A337SV16
Urine
AIATLMSDEVFHDIAYK
+3
tr|A0A2I2UF62
Urine
YVQELQK +2
tr|A0A2I2U0I8
Urine
FTGVITQGR +2
tr|A0A337SEH6
Urine
EAINVEQAFQTIAR +2
tr|A0A337SFP3
Urine
LGALSGAGALGLASYG
AHGAQFPDAYGK +3
tr|M3WSW8
Serum
ISSDFKIGQK +2
tr|A0A337S935
Urine
DIVQFVPFR +2
tr|A0A2I2U0K1
Urine
EGIHLALVELLK +3
tr|M3XDA8
Urine
VQEEKER +2
tr|A0A2I2V5H4
Urine
IYLDTAQISR +2
tr|A0A516V1E7
Urine
MALEVYK +2
tr|A0A5F5XEP4
Urine
WAWAPSFPACR +2
tr|A0A337S4A6
Urine
HQVTLPGQEEPWDIR
+3
tr|A0A5F5XS01
Urine
ILILNNNLLR +2
tr|M3VYI8
Urine
DRETVPPSAL +2
tr|M3WN82
Urine
VGEVFYTANR +2
tr|A0A5F5XQI4
Urine
LPELEQR +2
tr|A0A5F5XGS4
Plasma
AIENSARDAAVQK +3
tr|M3VZR2
Serum
GLEWLGYWSGSTTYNP
AFQGR +3
tr|A0A291NH02
Urine
VPTISINK +2
tr|A0A2I2U787
Urine
VAVLGASGGIGQPLSLL
LK +2
sp|K0J107
Urine
SSDGVVHASYDSR +3
tr|M3WAV8
MS2 (10^3)
MS1 (10^3)
MS1 (10^6)
MS2
MS1
Retention Time (min)

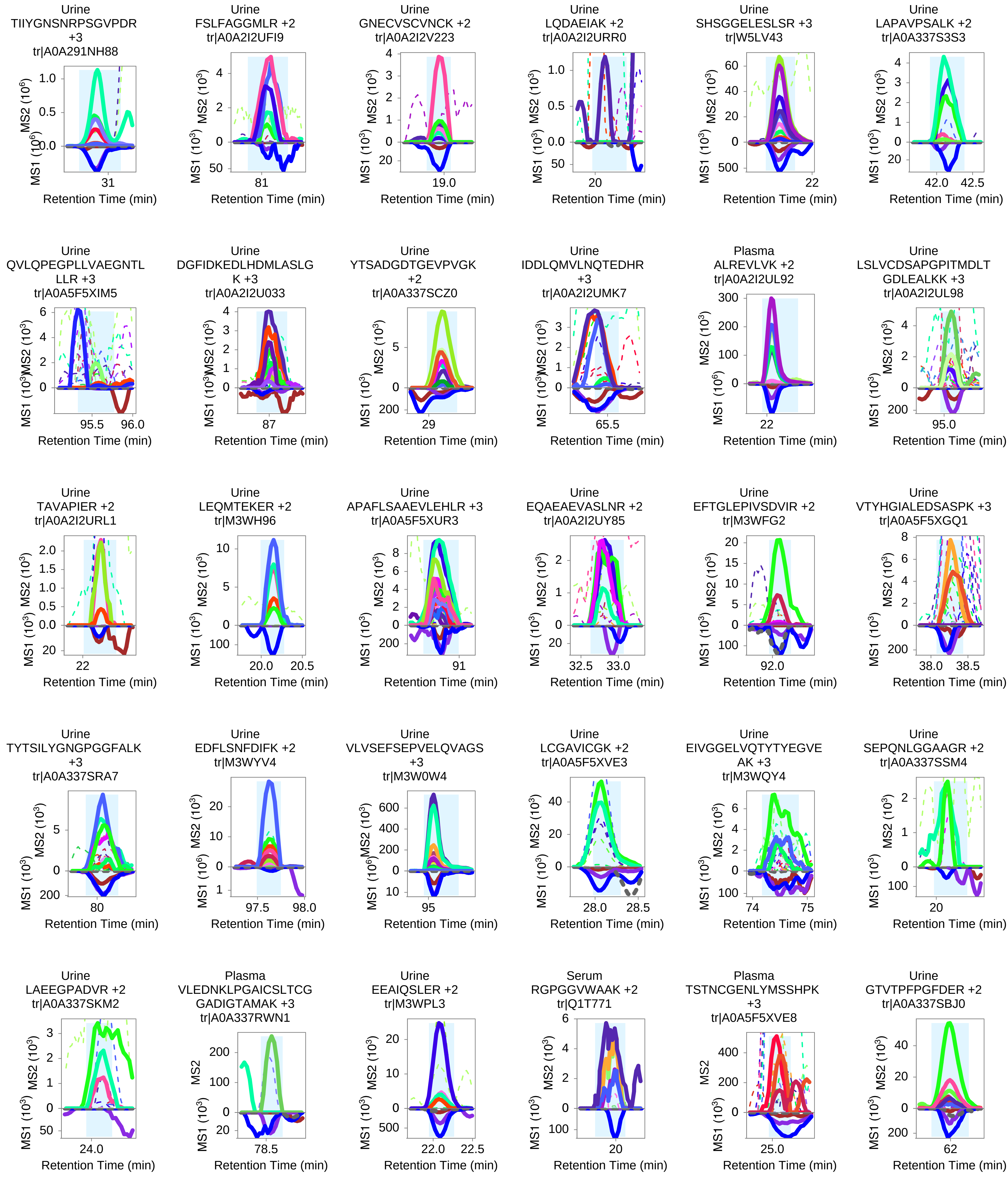

Urine
TIIYGNSNRPSGVPDR +3
tr|A0A291NH88
MS2 (10^6)
MS1 (10^6)
Retention Time (min)
Urine
FSLFAGGMLR +2
tr|A0A2I2UFI9
MS2 (10^3)
MS1 (10^3)
Retention Time (min)
Urine
GNECVSCVNCK +2
tr|A0A2I2V223
MS2 (10^3)
MS1 (10^3)
Retention Time (min)
Urine
LQDAEIAK +2
tr|A0A2I2URR0
MS2 (10^3)
MS1 (10^3)
Retention Time (min)
Urine
SHSGGELESLSR +3
tr|W5LV43
MS2 (10^3)
MS1 (10^3)
Retention Time (min)
Urine
LAPAVPSALK +2
tr|A0A337S3S3
MS2 (10^3)
MS1 (10^3)
Retention Time (min)
Urine
QVLQPEGPLLVAEGNTLLLR +3
tr|A0A5F5XIM5
MS2 (10^3)
MS1 (10^3)
Retention Time (min)
Urine
DGFIDKEDLHDMLASLGK +3
tr|A0A2I2U033
MS2 (10^3)
MS1 (10^3)
Retention Time (min)
Urine
YTSADGDTGEVPVGK +2
tr|A0A337SCZ0
MS2 (10^3)
MS1 (10^3)
Retention Time (min)
Urine
IDDLQMVLNQTEDHR +3
tr|A0A2I2UMK7
MS2 (10^3)
MS1 (10^3)
Retention Time (min)
Plasma
ALREVLVK +2
tr|A0A2I2UL92
MS2 (10^3)
MS1 (10^6)
Retention Time (min)
Urine
LSLVCDSAPGPITMDLTGDLEALKK +3
tr|A0A2I2UL98
MS2 (10^3)
MS1 (10^3)
Retention Time (min)
Urine
TAVAPIER +2
tr|A0A2I2URL1
MS2 (10^3)
MS1 (10^3)
Retention Time (min)
Urine
LEQMTEKER +2
tr|M3WH96
MS2 (10^3)
MS1 (10^3)
Retention Time (min)
Urine
APAFLSAAEVLEHLR +3
tr|A0A5F5XUR3
MS2 (10^3)
MS1 (10^3)
Retention Time (min)
Urine
EQAEAEVASLNR +2
tr|A0A2I2UY85
MS2 (10^3)
MS1 (10^3)
Retention Time (min)
Urine
EFTGLEPIVSDVIR +2
tr|M3WFG2
MS2 (10^3)
MS1 (10^3)
Retention Time (min)
Urine
VTYHGIALEDSASPK +3
tr|A0A5F5XGQ1
MS2 (10^3)
MS1 (10^3)
Retention Time (min)
Urine
TYTSILYGNGPGGFALK +3
tr|A0A337SRA7
MS2 (10^3)
MS1 (10^3)
Retention Time (min)
Urine
EDFLSNFDIFK +2
tr|M3WYV4
MS2 (10^3)
MS1 (10^6)
Retention Time (min)
Urine
VLVSEFSEPVELQVAGS +3
tr|M3W0W4
MS2 (10^3)
MS1 (10^6)
Retention Time (min)
Urine
LCGAVICGK +2
tr|A0A5F5XVE3
MS2 (10^3)
MS1 (10^3)
Retention Time (min)
Urine
EIVGGELVQTYTYEGVEAK +3
tr|M3WQY4
MS2 (10^3)
MS1 (10^3)
Retention Time (min)
Urine
SEPQNLGGAAGR +2
tr|A0A337SSM4
MS2 (10^3)
MS1 (10^3)
Retention Time (min)
Urine
LAEEGPADVR +2
tr|A0A337SKM2
MS2 (10^3)
MS1 (10^3)
Retention Time (min)
Plasma
VLEDNKLPGAICSLTCGGADIGTAMAK +3
tr|A0A337RWN1
MS2
MS1 (10^3)
Retention Time (min)
Urine
EEAIQSLER +2
tr|M3WPL3
MS2 (10^3)
MS1 (10^3)
Retention Time (min)
Serum
RGPGGVWAAK +2
tr|Q1T771
MS2 (10^3)
MS1 (10^3)
Retention Time (min)
Plasma
TSTNCGENLYMSSHPK +3
tr|A0A5F5XVE8
MS2
MS1 (10^3)
Retention Time (min)
Urine
GTVTPFPGFDER +2
tr|A0A337SBJ0
MS2 (10^3)
MS1 (10^3)
Retention Time (min)

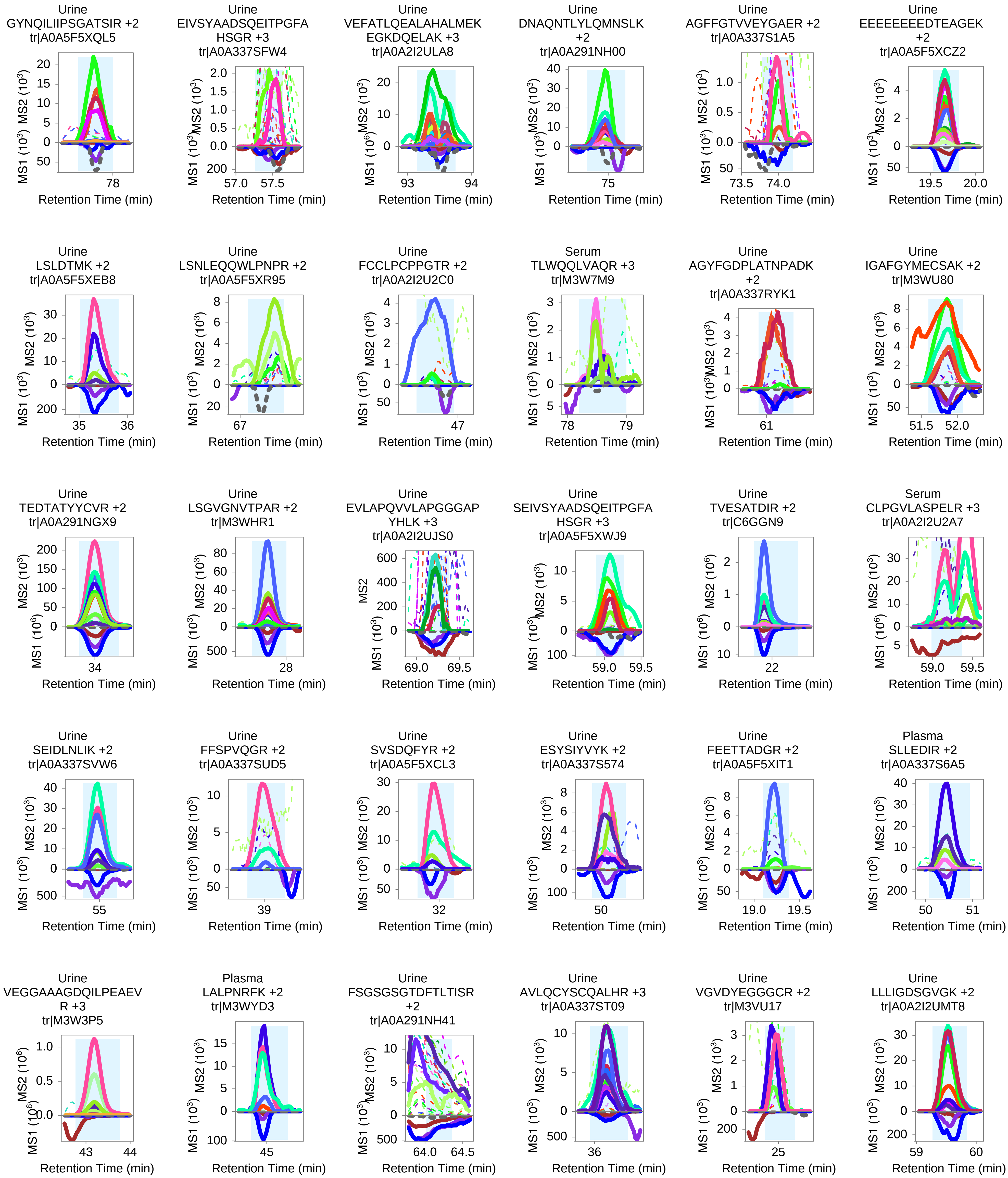

Urine
GYNQILIIPSGATSIR +2
tr|A0A5F5XQL5
Urine
EIVSYAADSQEITPGFA
HSGR +3
tr|A0A337SFW4
Urine
VEFATLQEALAHALMEK
EGKDQELAK +3
tr|A0A2I2ULA8
Urine
DNAQNTLYLQMNSLK
+2
tr|A0A291NH00
Urine
AGFFGTVVEYGAER +2
tr|A0A337S1A5
Urine
EEEEEEEEDTEAGEK
+2
tr|A0A5F5XCZ2
Urine
LSLDTMK +2
tr|A0A5F5XEB8
Urine
LSNLEQQWLPNPR +2
tr|A0A5F5XR95
Urine
FCCLPCPPGTR +2
tr|A0A2I2U2C0
Serum
TLWQQLVAQR +3
tr|M3W7M9
Urine
AGYFGDPLATNPADK
+2
tr|A0A337RYK1
Urine
IGAFGYMECSAK +2
tr|M3WU80
Urine
TEDTATYYCVR +2
tr|A0A291NGX9
Urine
LSGVGNVTPAR +2
tr|M3WHR1
Urine
EVLAPQVVLAPGGGAP
YHLK +3
tr|A0A2I2UJS0
Urine
SEIVSYAADSQEITPGFA
HSGR +3
tr|A0A5F5XWJ9
Urine
TVESATDIR +2
tr|C6GGN9
Serum
CLPGVLASPELR +3
tr|A0A2I2U2A7
Urine
SEIDLNLIK +2
tr|A0A337SVW6
Urine
FFSPVQGR +2
tr|A0A337SUD5
Urine
SVSDQFYR +2
tr|A0A5F5XCL3
Urine
ESYSIYVYK +2
tr|A0A337S574
Urine
FEETTADGR +2
tr|A0A5F5XIT1
Plasma
SLLEDIR +2
tr|A0A337S6A5
Urine
VEGGAAAGDQILPEAEV
R +3
tr|M3W3P5
Plasma
LALPNRFK +2
tr|M3WYD3
Urine
FSGSGSGTDFTLTISR
+2
tr|A0A291NH41
Urine
AVLQCYSCQALHR +3
tr|A0A337ST09
Urine
VGVDYEGGGCR +2
tr|M3VU17
Urine
LLLIGDSGVGK +2
tr|A0A2I2UMT8
MS2 (10^3)
MS2 (10^6)
MS2
MS1 (10^3)
MS1 (10^6)
Retention Time (min)

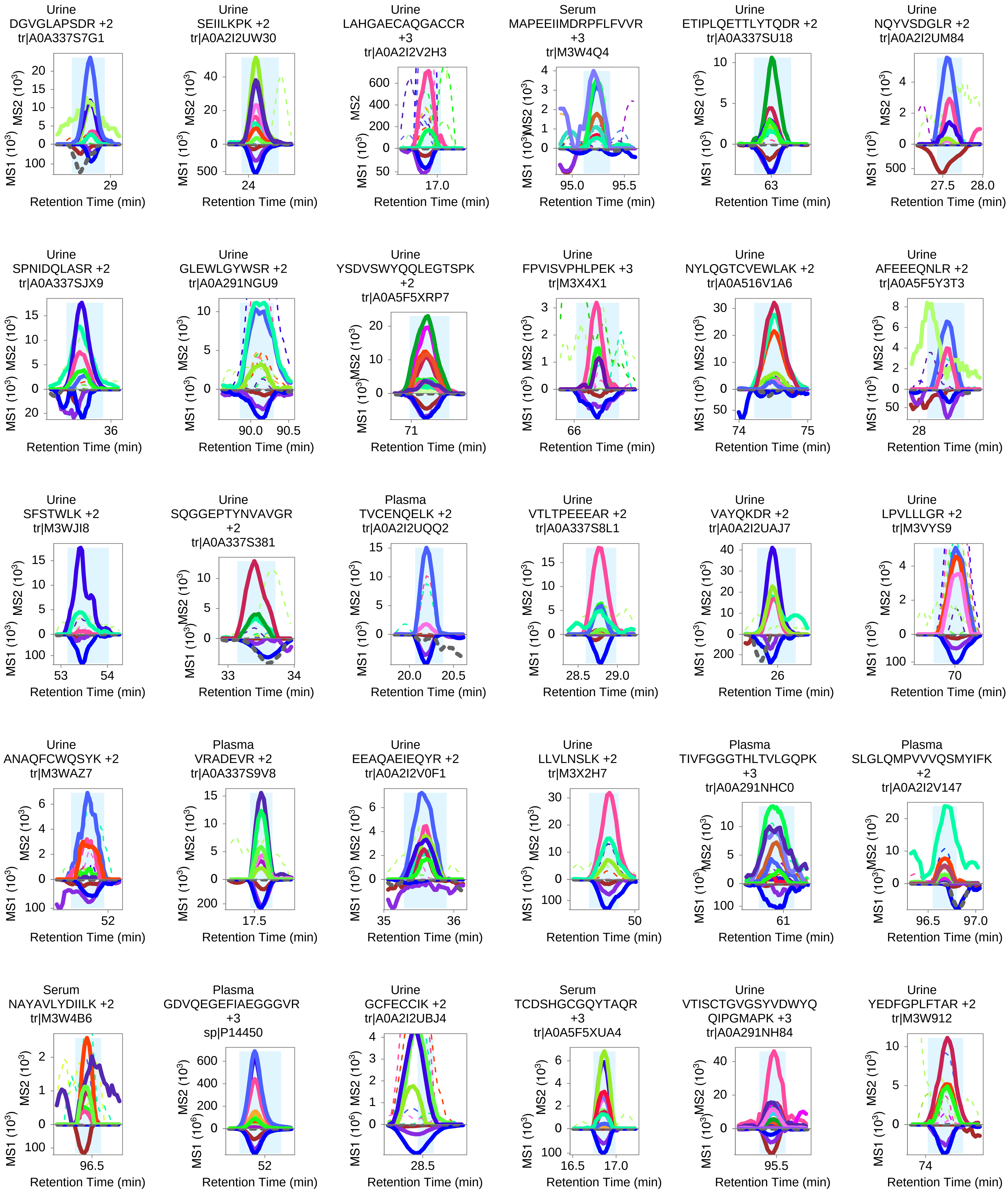
Urine
DGVGLAPSDR +2
tr|A0A337S7G1
Urine
SEIILKPK +2
tr|A0A2I2UW30
Urine
LAHGAECAQGACCR
+3
tr|A0A2I2V2H3
Serum
MAPEEIIMDRPFLFVVR
+3
tr|M3W4Q4
Urine
ETIPLQETTLYTQDR +2
tr|A0A337SU18
Urine
NQYVSDGLR +2
tr|A0A2I2UM84
Urine
SPNIDQLASR +2
tr|A0A337SJX9
Urine
GLEWLGYWSR +2
tr|A0A291NGU9
Urine
YSDVSWYQQLEGTSPK
+2
tr|A0A5F5XRP7
Urine
FPVISVPHLPEK +3
tr|M3X4X1
Urine
NYLQGTCVEWLAK +2
tr|A0A516V1A6
Urine
AFEEEQNLR +2
tr|A0A5F5Y3T3
Urine
SFSTWLK +2
tr|M3WJI8
Urine
SQGGEPTYNVAVGR
+2
tr|A0A337S381
Plasma
TVCENQELK +2
tr|A0A2I2UQQ2
Urine
VTLTPEEEAR +2
tr|A0A337S8L1
Urine
VAYQKDR +2
tr|A0A2I2UAJ7
Urine
LPVLLLGR +2
tr|M3VYS9
Urine
ANAQFCWQSYK +2
tr|M3WAZ7
Plasma
VRADEVR +2
tr|A0A337S9V8
Urine
EEAQAEIEQYR +2
tr|A0A2I2V0F1
Urine
LLVLNSLK +2
tr|M3X2H7
Plasma
TIVFGGGTHLTVLGQPK
+3
tr|A0A291NHC0
Plasma
SLGLQMPVVVQSMYIFK
+2
tr|A0A2I2V147
Serum
NAYAVLYDIILK +2
tr|M3W4B6
Plasma
GDVQEGEFIAEGGGVR
+3
sp|P14450
Urine
GCFECCIK +2
tr|A0A2I2UBJ4
Serum
TCDSHGCGQYTAQR
+3
tr|A0A5F5XUA4
Urine
VTISCTGVGSYVDWYQ
QIPGMAPK +3
tr|A0A291NH84
Urine
YEDFGPLFTAR +2
tr|M3W912
MS2 (10^3)
MS2
MS1 (10^3)
MS1 (10^6)
Retention Time (min)

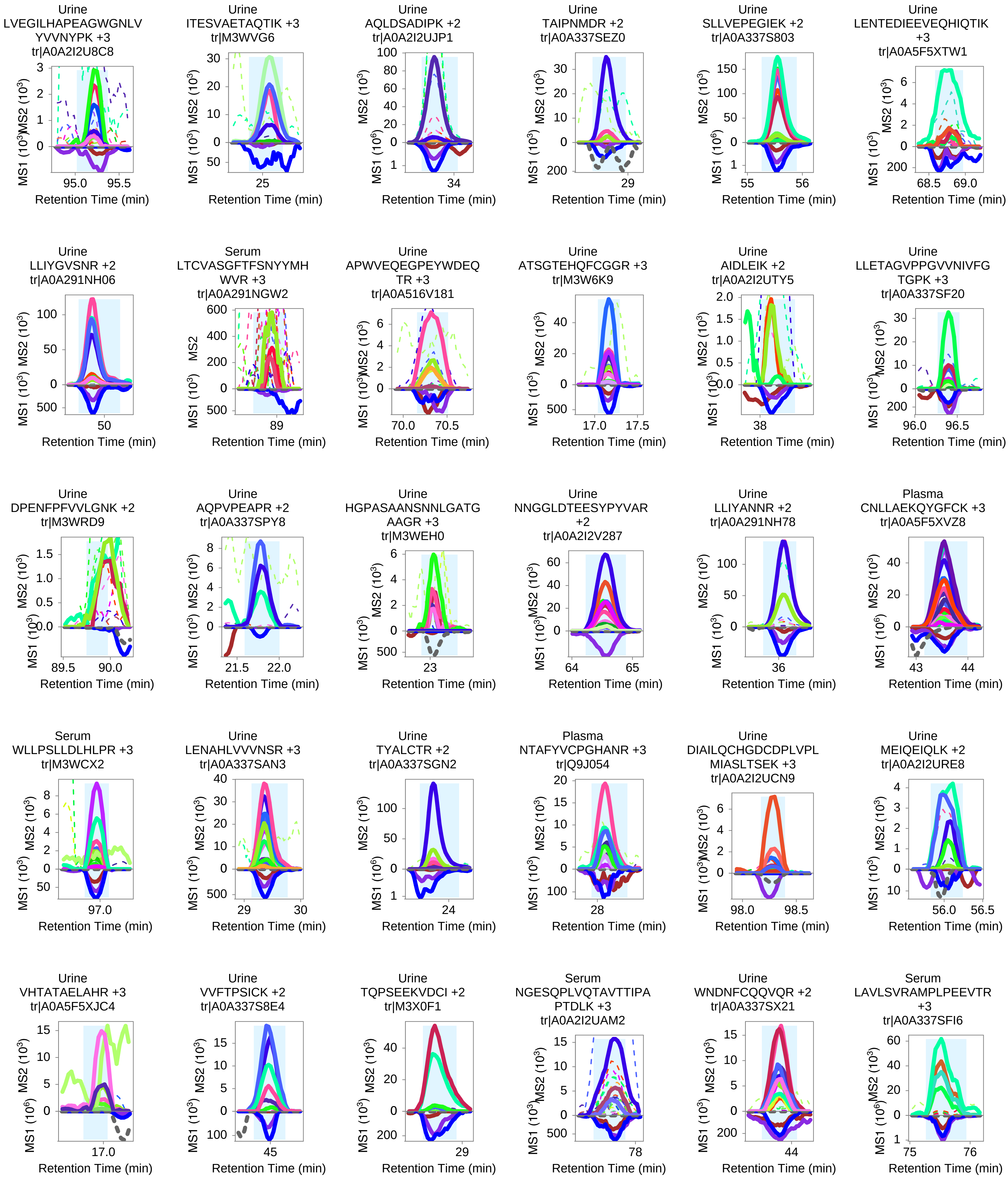
Urine
LVEGILHAPEAGWGNLV
YVVNYPK +3
tr|A0A2I2U8C8
Urine
ITESVAETAQTIK +3
tr|M3WVG6
Urine
AQLDSADIPK +2
tr|A0A2I2UJP1
Urine
TAIPNMDR +2
tr|A0A337SEZ0
Urine
SLLVEPEGIEK +2
tr|A0A337S803
Urine
LENTEDIEEVEQHIQTIK
+3
tr|A0A5F5XTW1
Urine
LLIYGVSNR +2
tr|A0A291NH06
Serum
LTCVASGFTFSNYYMH
WVR +3
tr|A0A291NGW2
Urine
APWVEQEGPEYWDEQ
TR +3
tr|A0A516V181
Urine
ATSGTEHQFCGGR +3
tr|M3W6K9
Urine
AIDLEIK +2
tr|A0A2I2UTY5
Urine
LLETAGVPPGVVNIVFG
TGPK +3
tr|A0A337SF20
Urine
DPENFPFVVLGNK +2
tr|M3WRD9
Urine
AQPVPEAPR +2
tr|A0A337SPY8
Urine
HGPASAANSNNLGATG
AAGR +3
tr|M3WEH0
Urine
NNGGLDTEESYPYVAR
+2
tr|A0A2I2V287
Urine
LLIYANNR +2
tr|A0A291NH78
Plasma
CNLLAEKQYGFCK +3
tr|A0A5F5XVZ8
Serum
WLLPSLLDLHLPR +3
tr|M3WCX2
Urine
LENAHLVVVNSR +3
tr|A0A337SAN3
Urine
TYALCTR +2
tr|A0A337SGN2
Plasma
NTAFYVCPGHANR +3
tr|Q9J054
Urine
DIAILQCHGDCDPLVPL
MIASLTSEK +3
tr|A0A2I2UCN9
Urine
MEIQEIQLK +2
tr|A0A2I2URE8
Urine
VHTATAELAHR +3
tr|A0A5F5XJC4
Urine
VVFTPSICK +2
tr|A0A337S8E4
Urine
TQPSEEKVDCI +2
tr|M3X0F1
Serum
NGESQPLVQTAVTTIPA
PTDLK +3
tr|A0A2I2UAM2
Urine
WNDNFCQQVQR +2
tr|A0A337SX21
Serum
LAVLSVRAMPLPEEVTR
+3
tr|A0A337SFI6
MS2 (10³)
MS1 (10³)
MS1 (10⁶)
Retention Time (min)

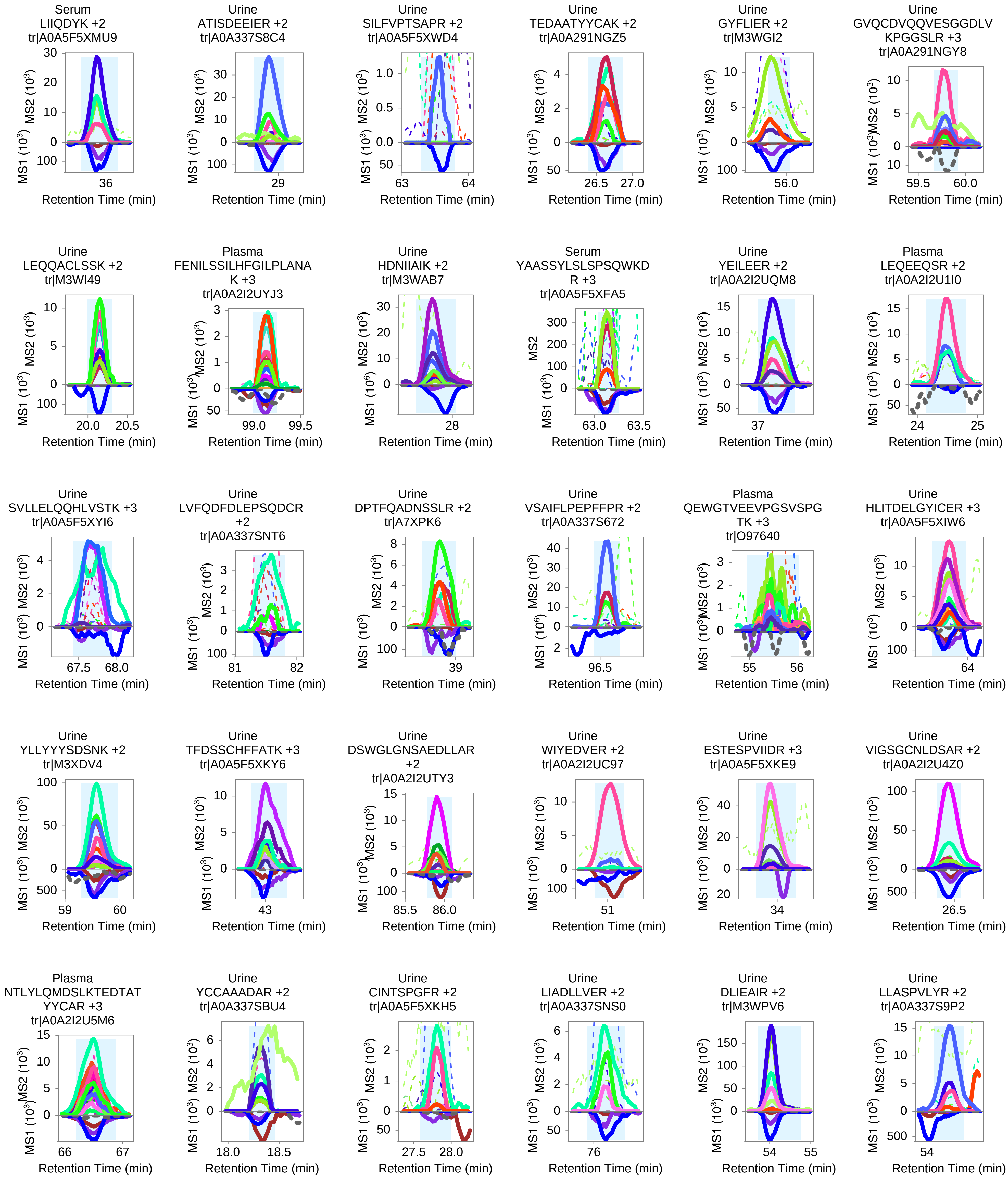
Serum
LIIQDYK +2
tr|A0A5F5XMU9
Urine
ATISDEEIER +2
tr|A0A337S8C4
Urine
SILFVPTSAPR +2
tr|A0A5F5XWD4
Urine
TEDAATYYCAK +2
tr|A0A291NGZ5
Urine
GYFLIER +2
tr|M3WGI2
Urine
GVQCDVQQVESGGDLV
KPGGSLR +3
tr|A0A291NGY8
Urine
LEQQACLSSK +2
tr|M3WI49
Plasma
FENILSSILHFGILPLANA
K +3
tr|A0A2I2UYJ3
Urine
HDNIIAIK +2
tr|M3WAB7
Serum
YAASSYLSLSPSQWKD
R +3
tr|A0A5F5XFA5
Urine
YEILEER +2
tr|A0A2I2UQM8
Plasma
LEQEEQSR +2
tr|A0A2I2U1I0
Urine
SVLLELQQHLVSTK +3
tr|A0A5F5XYI6
Urine
LVFQDFDLEPSQDCR
+2
tr|A0A337SNT6
Urine
DPTFQADNSSLR +2
tr|A7XPK6
Urine
VSAIFLPEPFFPR +2
tr|A0A337S672
Plasma
QEWGTVEEVPGSVSPG
TK +3
tr|O97640
Urine
HLITDELGYICER +3
tr|A0A5F5XIW6
Urine
YLLYYYSDSNK +2
tr|M3XDV4
Urine
TFDSSCHFFATK +3
tr|A0A5F5XKY6
Urine
DSWGLGNSAEDLLAR
+2
tr|A0A2I2UTY3
Urine
WIYEDVER +2
tr|A0A2I2UC97
Urine
ESTESPVIIDR +3
tr|A0A5F5XKE9
Urine
VIGSGCNLDSAR +2
tr|A0A2I2U4Z0
Plasma
NTLYLQMDSLKTEDTAT
YYCAR +3
tr|A0A2I2U5M6
Urine
YCCAAADAR +2
tr|A0A337SBU4
Urine
CINTSPGFR +2
tr|A0A5F5XKH5
Urine
LIADLLVER +2
tr|A0A337SNS0
Urine
DLIEAIR +2
tr|M3WPV6
Urine
LLASPVLYR +2
tr|A0A337S9P2
MS1 (10³) MS2 (10³)
Retention Time (min)

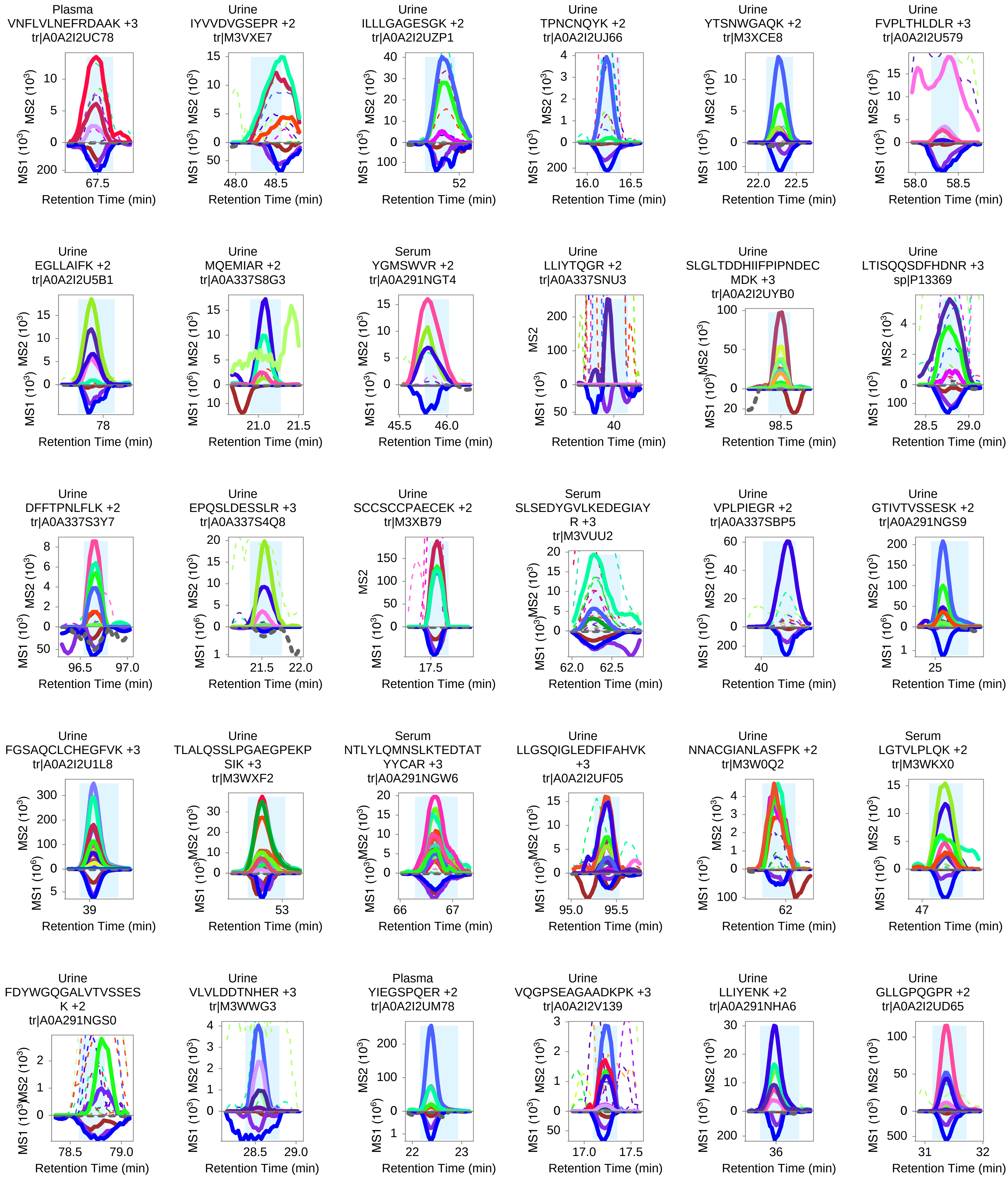
Plasma
VNFLVLNEFRDAAK +3
tr|A0A2I2UC78
Urine
IYVVDVGSEPR +2
tr|M3VXE7
Urine
ILLLGAGESGK +2
tr|A0A2I2UZP1
Urine
TPNCNQYK +2
tr|A0A2I2UJ66
Urine
YTSNWGAQK +2
tr|M3XCE8
Urine
FVPLTHLDLR +3
tr|A0A2I2U579
Urine
EGLLAIFK +2
tr|A0A2I2U5B1
Urine
MQEMIAR +2
tr|A0A337S8G3
Serum
YGMSWVR +2
tr|A0A291NGT4
Urine
LLIYTQGR +2
tr|A0A337SNU3
Urine
SLGLTDDHIIFPIPNDEC
MDK +3
tr|A0A2I2UYB0
Urine
LTISQQSDFHDNR +3
sp|P13369
Urine
DFFTPNLFLK +2
tr|A0A337S3Y7
Urine
EPQSLDESSLR +3
tr|A0A337S4Q8
Urine
SCCSCCPAECEK +2
tr|M3XB79
Serum
SLSEDYGVLKEDEGIAY
R +3
tr|M3VUU2
Urine
VPLPIEGR +2
tr|A0A337SBP5
Urine
GTIVTVSSESK +2
tr|A0A291NGS9
Urine
FGSAQCLCHEGFVK +3
tr|A0A2I2U1L8
Urine
TLALQSSLPGAEGPEKP
SIK +3
tr|M3WXF2
Serum
NTLYLQMNSLKTEDTAT
YYCAR +3
tr|A0A291NGW6
Urine
LLGSQIGLEDFIFAHVK
+3
tr|A0A2I2UF05
Urine
NNACGIANLASFPK +2
tr|M3W0Q2
Serum
LGTVLPLQK +2
tr|M3WKX0
Urine
FDYWGQGALVTVSSES
K +2
tr|A0A291NGS0
Urine
VLVLDDTNHER +3
tr|M3WWG3
Plasma
YIEGSPQER +2
tr|A0A2I2UM78
Urine
VQGPSEAGAADKPK +3
tr|A0A2I2V139
Urine
LLIYENK +2
tr|A0A291NHA6
Urine
GLLGPQGPR +2
tr|A0A2I2UD65
MS2 (10³)
MS1 (10³)
Retention Time (min)

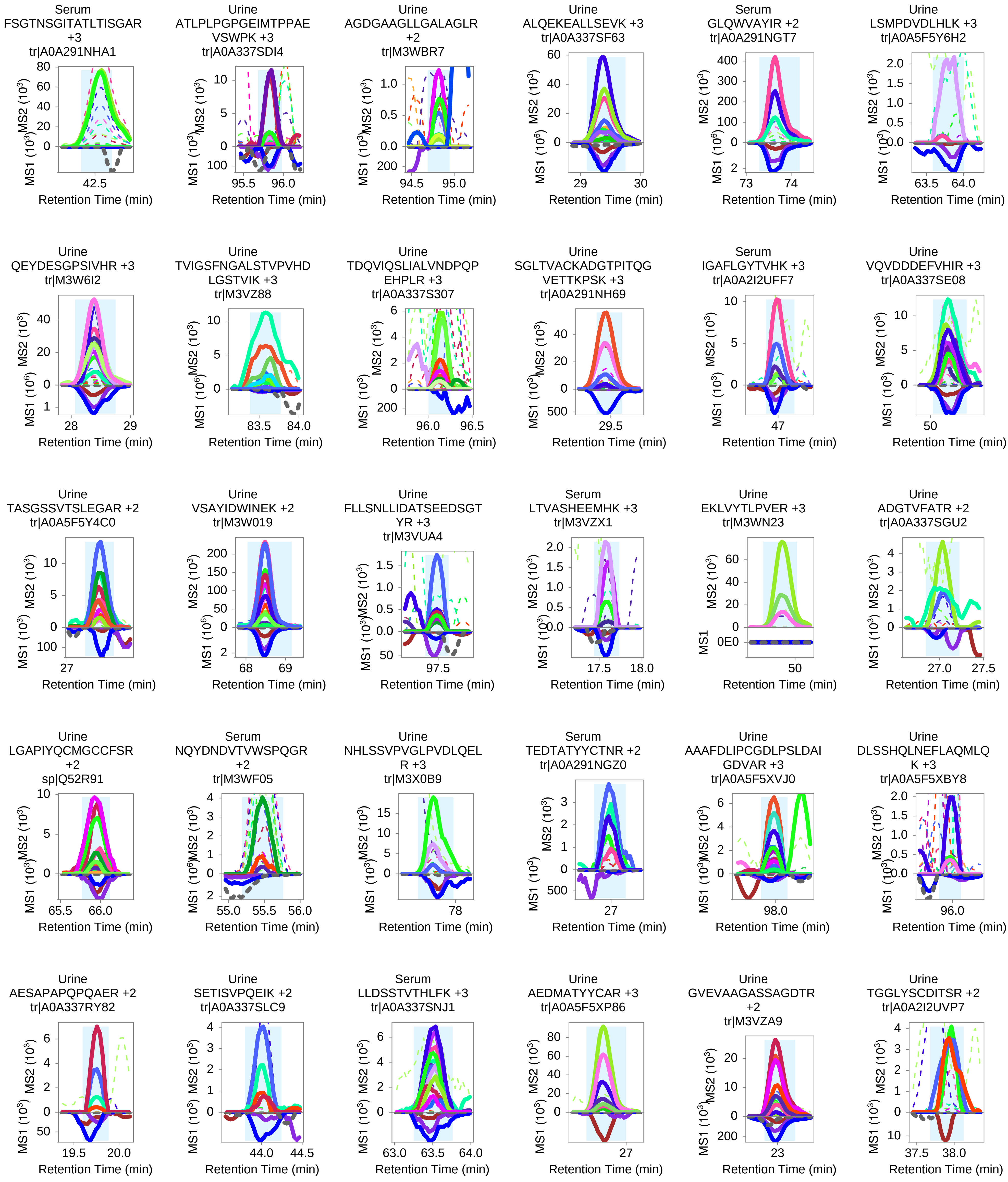

Serum
FSGTNSGITATLTISGAR +3
tr|A0A291NHA1
Urine
ATLPLPGPGEIMTPPAE VSWPK +3
tr|A0A337SDI4
Urine
AGDGAAGLLGALAGLR +2
tr|M3WBR7
Urine
ALQEKEALLSEVK +3
tr|A0A337SF63
Serum
GLQWVAYIR +2
tr|A0A291NGT7
Urine
LSMPDVDLHLK +3
tr|A0A5F5Y6H2
Urine
QEYDESGPSIVHR +3
tr|M3W6I2
Urine
TVIGSFNGALSTVPVHD LGSTVIK +3
tr|M3VZ88
Urine
TDQVIQSLIALVNDPQP EHPLR +3
tr|A0A337S307
Urine
SGLTVACKADGTPITQG VETTKPSK +3
tr|A0A291NH69
Serum
IGAFLGYTVHK +3
tr|A0A2I2UFF7
Urine
VQVDDDEFVHIR +3
tr|A0A337SE08
Urine
TASGSSVTSLEGAR +2
tr|A0A5F5Y4C0
Urine
VSAYIDWINEK +2
tr|M3W019
Urine
FLLSNLLIDATSEEDSGT YR +3
tr|M3VUA4
Serum
LTVASHEEMHK +3
tr|M3VZX1
Urine
EKLVYTLPVER +3
tr|M3WN23
Urine
ADGTVFATR +2
tr|A0A337SGU2
Urine
LGAPIYQCMGCCFSR +2
sp|Q52R91
Serum
NQYDNDVTVWSPQGR +2
tr|M3WF05
Urine
NHLSSVPVGLPVDLQEL R +3
tr|M3X0B9
Serum
TEDTATYYCTNR +2
tr|A0A291NGZ0
Urine
AAAFDLIPCGDLPSLDAI GDVAR +3
tr|A0A5F5XVJ0
Urine
DLSSHQLNEFLAQMLQ K +3
tr|A0A5F5XBY8
Urine
AESAPAPQPQAER +2
tr|A0A337RY82
Urine
SETISVPQEIK +2
tr|A0A337SLC9
Serum
LLDSSTVTHLFK +3
tr|A0A337SNJ1
Urine
AEDMATYYCAR +3
tr|A0A5F5XP86
Urine
GVEVAAGASSAGDTR +2
tr|M3VZA9
Urine
TGGLYSCDITSR +2
tr|A0A2I2UVP7
MS2 (10³)
MS1 (10³)
MS1 (10⁶)
Retention Time (min)

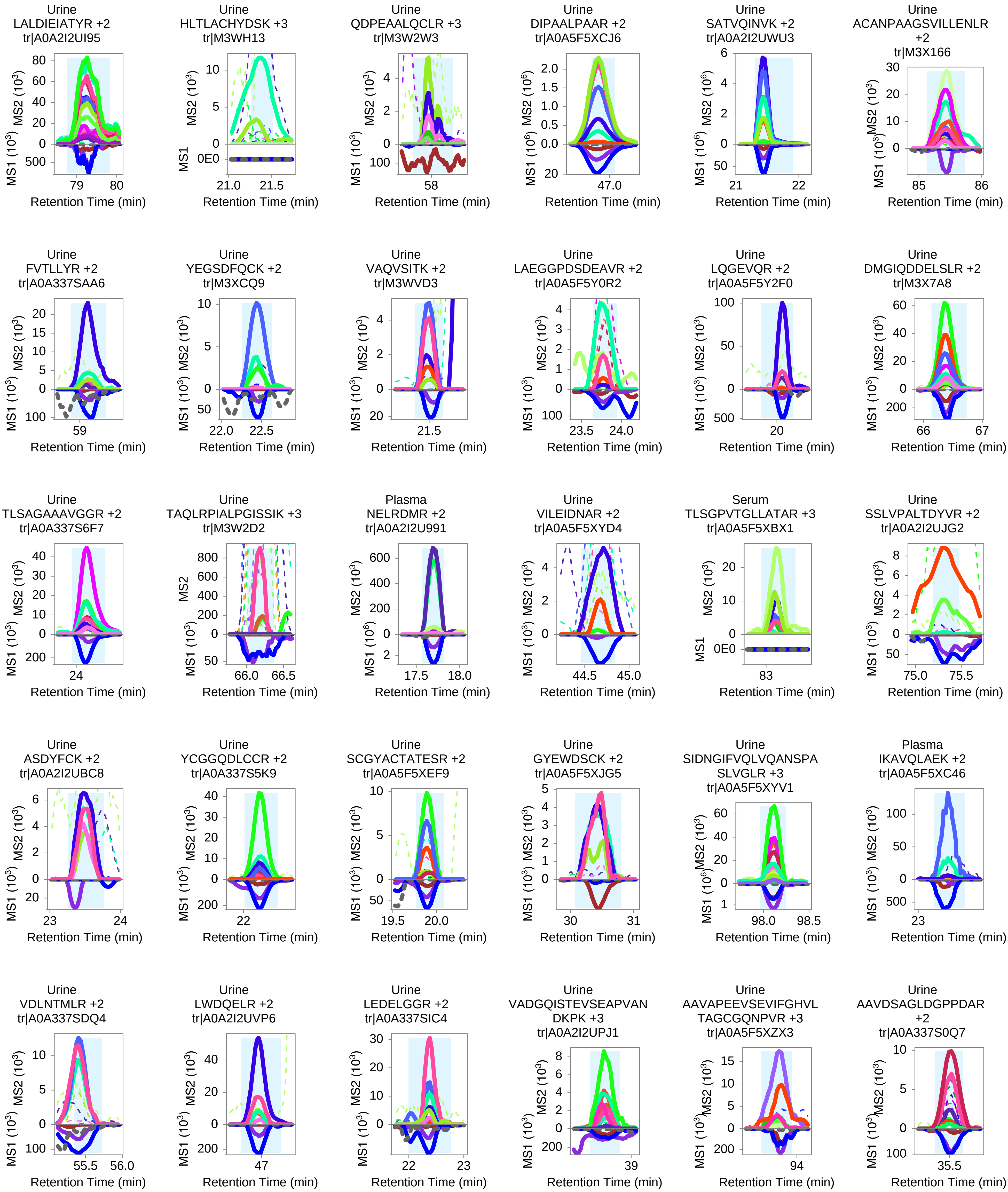
Urine
LALDIEIATYR +2
tr|A0A2I2UI95
MS1 (10³) MS2 (10³)
Retention Time (min)
Urine
HLTLACHYDSK +3
tr|M3WH13
MS1 MS2 (10³)
0E0
Retention Time (min)
Urine
QDPEAALQCLR +3
tr|M3W2W3
MS1 (10³) MS2 (10³)
Retention Time (min)
Urine
DIPAALPAAR +2
tr|A0A5F5XCJ6
MS1 (10⁶) MS2 (10⁶)
Retention Time (min)
Urine
SATVQINVK +2
tr|A0A2I2UWU3
MS1 (10⁶) MS2 (10⁶)
Retention Time (min)
Urine
ACANPAAGSVILLENLR +2
tr|M3X166
MS1 (10³) MS2 (10³)
Retention Time (min)
Urine
FVTLLYR +2
tr|A0A337SAA6
MS1 (10³) MS2 (10³)
Retention Time (min)
Urine
YEGSDFQCK +2
tr|M3XCQ9
MS1 (10³) MS2 (10³)
Retention Time (min)
Urine
VAQVSITK +2
tr|M3WVD3
MS1 (10³) MS2 (10³)
Retention Time (min)
Urine
LAEGGPDSDEAVR +2
tr|A0A5F5Y0R2
MS1 (10³) MS2 (10³)
Retention Time (min)
Urine
LQGEVQR +2
tr|A0A5F5Y2F0
MS1 (10³) MS2 (10³)
Retention Time (min)
Urine
DMGIQDDELSLR +2
tr|M3X7A8
MS1 (10³) MS2 (10³)
Retention Time (min)
Urine
TLSAGAAAVGGR +2
tr|A0A337S6F7
MS1 (10³) MS2 (10³)
Retention Time (min)
Urine
TAQLRPIALPGISSIK +3
tr|M3W2D2
MS1 (10³) MS2
Retention Time (min)
Plasma
NELRDMR +2
tr|A0A2I2U991
MS1 (10⁶) MS2 (10³)
Retention Time (min)
Urine
VILEIDNAR +2
tr|A0A5F5XYD4
MS1 (10³) MS2 (10³)
Retention Time (min)
Serum
TLSGPVTGLLATAR +3
tr|A0A5F5XBX1
MS1 MS2 (10³)
0E0
Retention Time (min)
Urine
SSLVPALTDYVR +2
tr|A0A2I2UJG2
MS1 (10³) MS2 (10³)
Retention Time (min)
Urine
ASDYFCK +2
tr|A0A2I2UBC8
MS1 (10³) MS2 (10³)
Retention Time (min)
Urine
YCGGQDLCCR +2
tr|A0A337S5K9
MS1 (10³) MS2 (10³)
Retention Time (min)
Urine
SCGYACTATESR +2
tr|A0A5F5XEF9
MS1 (10³) MS2 (10³)
Retention Time (min)
Urine
GYEWDSCK +2
tr|A0A5F5XJG5
MS1 (10³) MS2 (10³)
Retention Time (min)
Urine
SIDNGIFVQLVQANSPA
SLVGLR +3
tr|A0A5F5XYV1
MS1 (10⁶) MS2 (10³)
Retention Time (min)
Plasma
IKAVQLAEK +2
tr|A0A5F5XC46
MS1 (10³) MS2 (10³)
Retention Time (min)
Urine
VDLNTMLR +2
tr|A0A337SDQ4
MS1 (10³) MS2 (10³)
Retention Time (min)
Urine
LWDQELR +2
tr|A0A2I2UVP6
MS1 (10³) MS2 (10³)
Retention Time (min)
Urine
LEDELGGR +2
tr|A0A337SIC4
MS1 (10³) MS2 (10³)
Retention Time (min)
Urine
VADGQISTEVSEAPVAN
DKPK +3
tr|A0A2I2UPJ1
MS1 (10³) MS2 (10³)
Retention Time (min)
Urine
AAVAPEEVSEVIFGHVL
TAGCGQNPVR +3
tr|A0A5F5XZX3
MS1 (10³) MS2 (10³)
Retention Time (min)
Urine
AAVDSAGLDGPPDAR
+2
tr|A0A337S0Q7
MS1 (10³) MS2 (10³)
Retention Time (min)

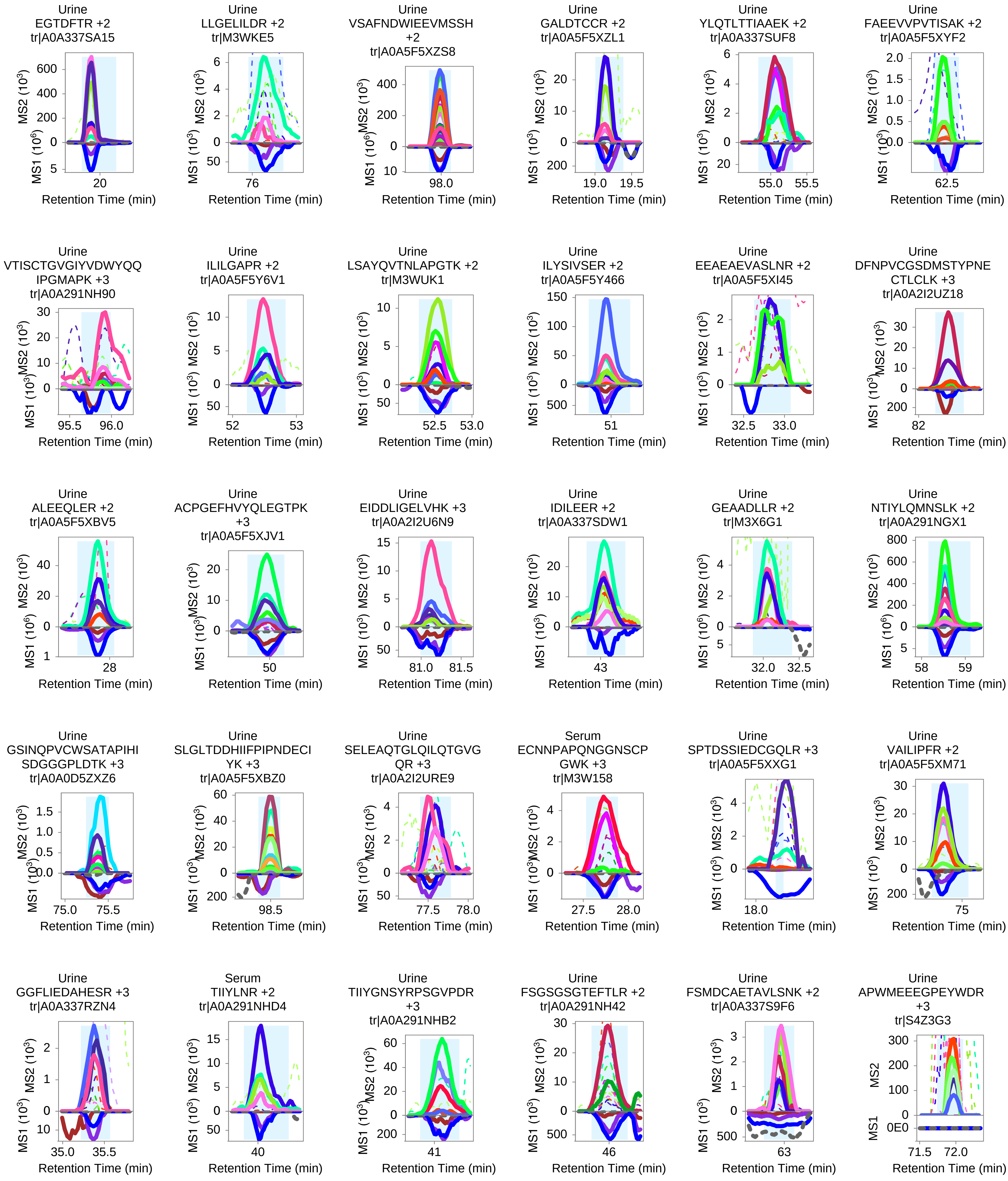
Urine
EGTDFTR +2
tr|A0A337SA15
Urine
LLGELILDR +2
tr|M3WKE5
Urine
VSAFNDWIEEVMSSH
+2
tr|A0A5F5XZS8
Urine
GALDTCCR +2
tr|A0A5F5XZL1
Urine
YLQTLTTIAAEK +2
tr|A0A337SUF8
Urine
FAEEVVPVTISAK +2
tr|A0A5F5XYF2
Urine
VTISCTGVGIYVDWYQQ
IPGMAPK +3
tr|A0A291NH90
Urine
ILILGAPR +2
tr|A0A5F5Y6V1
Urine
LSAYQVTNLAPGTK +2
tr|M3WUK1
Urine
ILYSIVSER +2
tr|A0A5F5Y466
Urine
EEAEAEVASLNR +2
tr|A0A5F5XI45
Urine
DFNPVCGSDMSTYPNE
CTLCLK +3
tr|A0A2I2UZ18
Urine
ALEEQLER +2
tr|A0A5F5XBV5
Urine
ACPGEFHVYQLEGTPK
+3
tr|A0A5F5XJV1
Urine
EIDDLIGELVHK +3
tr|A0A2I2U6N9
Urine
IDILEER +2
tr|A0A337SDW1
Urine
GEAADLLR +2
tr|M3X6G1
Urine
NTIYLQMNSLK +2
tr|A0A291NGX1
Urine
GSINQPVCWSATAPIHI
SDGGGPLDTK +3
tr|A0A0D5ZXZ6
Urine
SLGLTDDHIIFPIPNDECI
YK +3
tr|A0A5F5XBZ0
Urine
SELEAQTGLQILQTGVG
QR +3
tr|A0A2I2URE9
Serum
ECNNPAPQNGGNSCP
GWK +3
tr|M3W158
Urine
SPTDSSIEDCGQLR +3
tr|A0A5F5XXG1
Urine
VAILIPFR +2
tr|A0A5F5XM71
Urine
GGFLIEDAHESR +3
tr|A0A337RZN4
Serum
TIIYLNR +2
tr|A0A291NHD4
Urine
TIIYGNSYRPSGVPDR
+3
tr|A0A291NHB2
Urine
FSGSGSGTEFTLR +2
tr|A0A291NH42
Urine
FSMDCAETAVLSNK +2
tr|A0A337S9F6
Urine
APWMEEEGPEYWDR
+3
tr|S4Z3G3
MS2 (10³)
MS1 (10⁶)
MS1 (10³)
Retention Time (min)

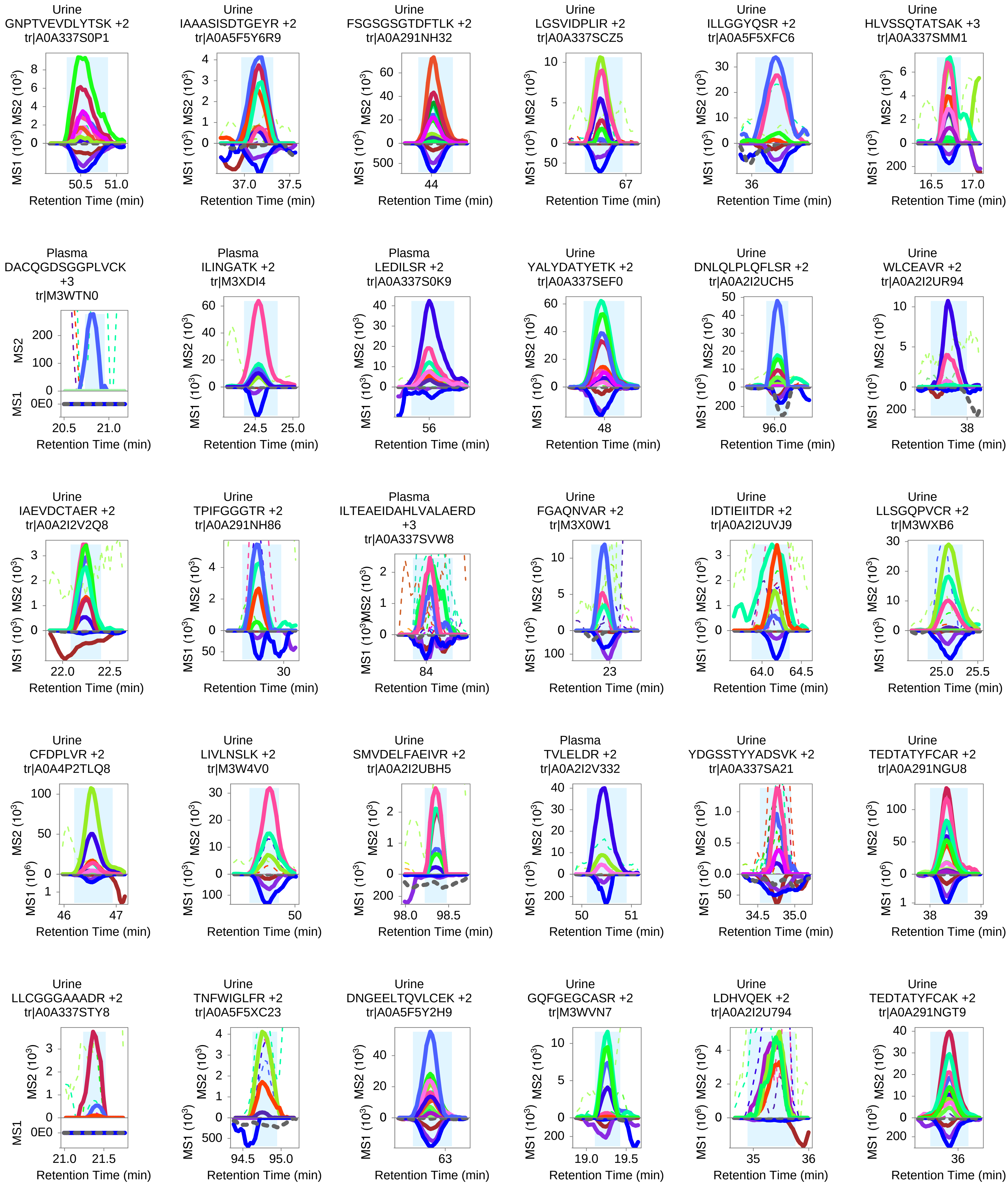
Urine
GNPTVEVDLYTSK +2
tr|A0A337S0P1
MS1 (10³) MS2 (10³)
8
6
4
2
0
50.5
51.0
Retention Time (min)
Urine
IAAASISDTGEYR +2
tr|A0A5F5Y6R9
MS1 (10³) MS2 (10³)
4
3
2
1
0
37.0
37.5
Retention Time (min)
Urine
FSGSGSGTDFTLK +2
tr|A0A291NH32
MS1 (10³) MS2 (10³)
60
40
20
0
500
44
Retention Time (min)
Urine
LGSVIDPLIR +2
tr|A0A337SCZ5
MS1 (10³) MS2 (10³)
10
5
0
50
67
Retention Time (min)
Urine
ILLGGYQSR +2
tr|A0A5F5XFC6
MS1 (10³) MS2 (10³)
30
20
10
0
36
Retention Time (min)
Urine
HLVSSQTATSAK +3
tr|A0A337SMM1
MS1 (10³) MS2 (10³)
6
4
2
0
200
16.5
17.0
Retention Time (min)
Plasma
DACQGDSGGPLVCK
+3
tr|M3WTN0
MS1 MS2
200
100
0
0E0
20.5
21.0
Retention Time (min)
Plasma
ILINGATK +2
tr|M3XDI4
MS1 (10³) MS2 (10³)
60
40
20
0
24.5
25.0
Retention Time (min)
Plasma
LEDILSR +2
tr|A0A337S0K9
MS1 (10³) MS2 (10³)
40
30
20
10
0
56
Retention Time (min)
Urine
YALYDATYETK +2
tr|A0A337SEF0
MS1 (10³) MS2 (10³)
60
40
20
0
48
Retention Time (min)
Urine
DNLQLPLQFLSR +2
tr|A0A2I2UCH5
MS1 (10³) MS2 (10³)
50
40
30
20
10
0
200
96.0
Retention Time (min)
Urine
WLCEAVR +2
tr|A0A2I2UR94
MS1 (10³) MS2 (10³)
10
5
0
200
38
Retention Time (min)
Urine
IAEVDCTAER +2
tr|A0A2I2V2Q8
MS1 (10³) MS2 (10³)
3
2
1
0
22.0
22.5
Retention Time (min)
Urine
TPIFGGGTR +2
tr|A0A291NH86
MS1 (10³) MS2 (10³)
4
2
0
50
30
Retention Time (min)
Plasma
ILTEAEIDAHLVALAERD
+3
tr|A0A337SVW8
MS1 (10³) MS2 (10³)
2
1
0
84
Retention Time (min)
Urine
FGAQNVAR +2
tr|M3X0W1
MS1 (10³) MS2 (10³)
10
5
0
100
23
Retention Time (min)
Urine
IDTIEIITDR +2
tr|A0A2I2UVJ9
MS1 (10³) MS2 (10³)
3
2
1
0
64.0
64.5
Retention Time (min)
Urine
LLSGQPVCR +2
tr|M3WXB6
MS1 (10³) MS2 (10³)
30
20
10
0
25.0
25.5
Retention Time (min)
Urine
CFDPLVR +2
tr|A0A4P2TLQ8
MS1 (10⁶) MS2 (10³)
100
50
0
1
46
47
Retention Time (min)
Urine
LIVLNSLK +2
tr|M3W4V0
MS1 (10³) MS2 (10³)
30
20
10
0
100
50
Retention Time (min)
Urine
SMVDELFAEIVR +2
tr|A0A2I2UBH5
MS1 (10³) MS2 (10³)
2
1
0
200
98.0
98.5
Retention Time (min)
Plasma
TVLELDR +2
tr|A0A2I2V332
MS1 (10³) MS2 (10³)
40
30
20
10
0
200
50
51
Retention Time (min)
Urine
YDGSSTYYADSVK +2
tr|A0A337SA21
MS1 (10³) MS2 (10³)
1.0
0.5
0.0
50
34.5
35.0
Retention Time (min)
Urine
TEDTATYFCAR +2
tr|A0A291NGU8
MS1 (10⁶) MS2 (10³)
100
50
0
1
38
39
Retention Time (min)
Urine
LLCGGGAAADR +2
tr|A0A337STY8
MS1 MS2 (10³)
3
2
1
0
0E0
21.0
21.5
Retention Time (min)
Urine
TNFWIGLFR +2
tr|A0A5F5XC23
MS1 (10³) MS2 (10³)
4
3
2
1
0
500
94.5
95.0
Retention Time (min)
Urine
DNGEELTQVLCEK +2
tr|A0A5F5Y2H9
MS1 (10³) MS2 (10³)
40
20
0
63
Retention Time (min)
Urine
GQFGEGCASR +2
tr|M3WVN7
MS1 (10³) MS2 (10³)
10
5
0
200
19.0
19.5
Retention Time (min)
Urine
LDHVQEK +2
tr|A0A2I2U794
MS1 (10⁶) MS2 (10³)
4
2
0
35
36
Retention Time (min)
Urine
TEDTATYFCAK +2
tr|A0A291NGT9
MS1 (10³) MS2 (10³)
40
30
20
10
0
200
36
Retention Time (min)

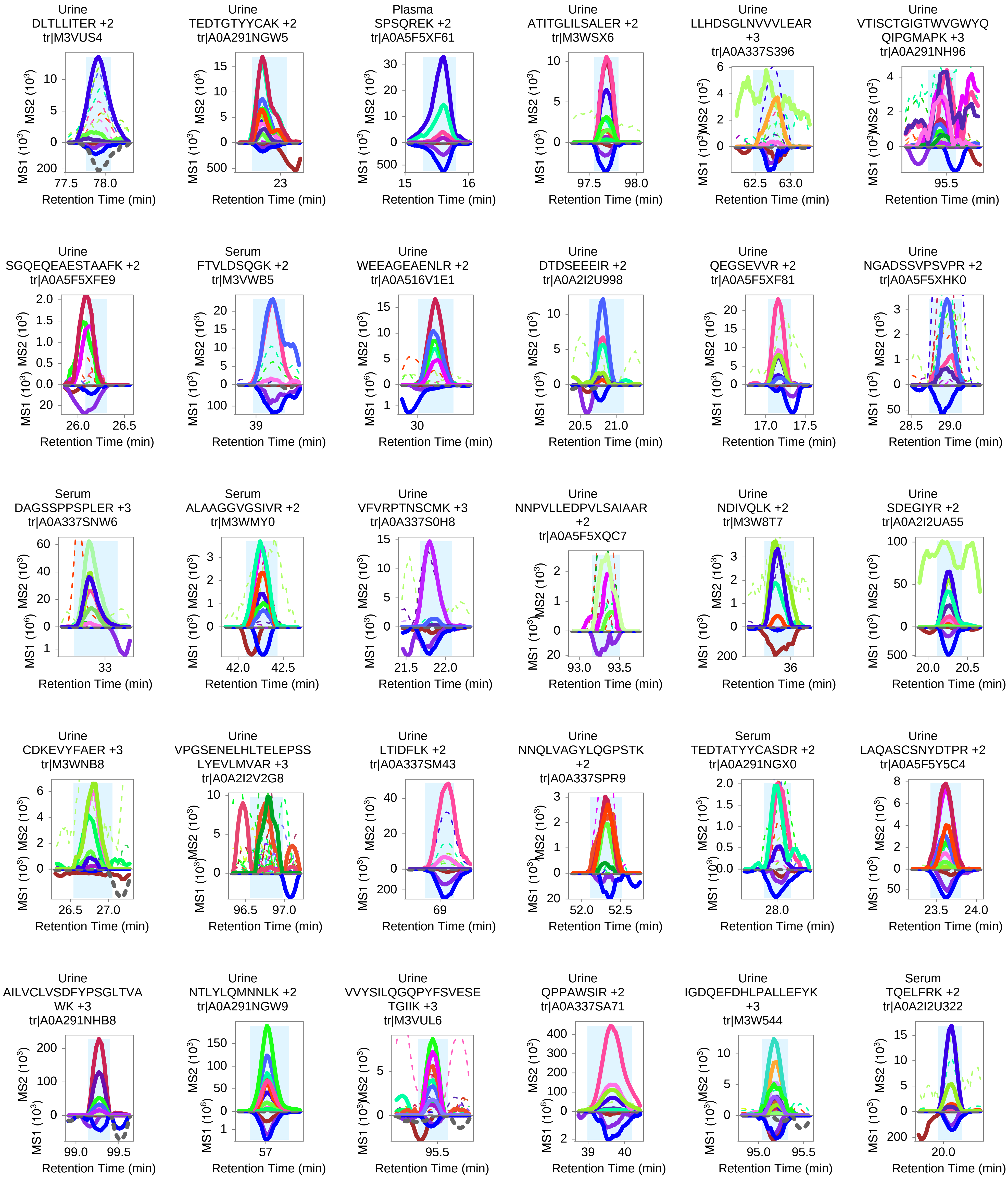

Urine
DLTLLITER +2
tr|M3VUS4
Urine
TEDTGTYYCAK +2
tr|A0A291NGW5
Plasma
SPSQREK +2
tr|A0A5F5XF61
Urine
ATITGLILSALER +2
tr|M3WSX6
Urine
LLHDSGLNVVVLEAR +3
tr|A0A337S396
Urine
VTISCTGIGTWVGWYQ QIPGMAPK +3
tr|A0A291NH96
Urine
SGQEQEAESTAAFK +2
tr|A0A5F5XFE9
Serum
FTVLDSQGK +2
tr|M3VWB5
Urine
WEEAGEAENLR +2
tr|A0A516V1E1
Urine
DTDSEEEIR +2
tr|A0A2I2U998
Urine
QEGSEVVR +2
tr|A0A5F5XF81
Urine
NGADSSVPSVPR +2
tr|A0A5F5XHK0
Serum
DAGSSPPSPLER +3
tr|A0A337SNW6
Serum
ALAAGGVGSIVR +2
tr|M3WMY0
Urine
VFVRPTNSCMK +3
tr|A0A337S0H8
Urine
NNPVLLEDPVLSAIAAR +2
tr|A0A5F5XQC7
Urine
NDIVQLK +2
tr|M3W8T7
Urine
SDEGIYR +2
tr|A0A2I2UA55
Urine
CDKEVYFAER +3
tr|M3WNB8
Urine
VPGSENELHLTELEPSS LYEVLMVAR +3
tr|A0A2I2V2G8
Urine
LTIDFLK +2
tr|A0A337SM43
Urine
NNQLVAGYLQGPSTK +2
tr|A0A337SPR9
Serum
TEDTATYYCASDR +2
tr|A0A291NGX0
Urine
LAQASCSNYDTPR +2
tr|A0A5F5Y5C4
Urine
AILVCLVSDFYPSGLTVA WK +3
tr|A0A291NHB8
Urine
NTLYLQMNNLK +2
tr|A0A291NGW9
Urine
VVYSILQGQPYFSVESE TGIIK +3
tr|M3VUL6
Urine
QPPAWSIR +2
tr|A0A337SA71
Urine
IGDQEFDHLPALLEFYK +3
tr|M3W544
Serum
TQELFRK +2
tr|A0A2I2U322
MS2 (10³)
MS1 (10³)
MS1 (10⁶)
Retention Time (min)

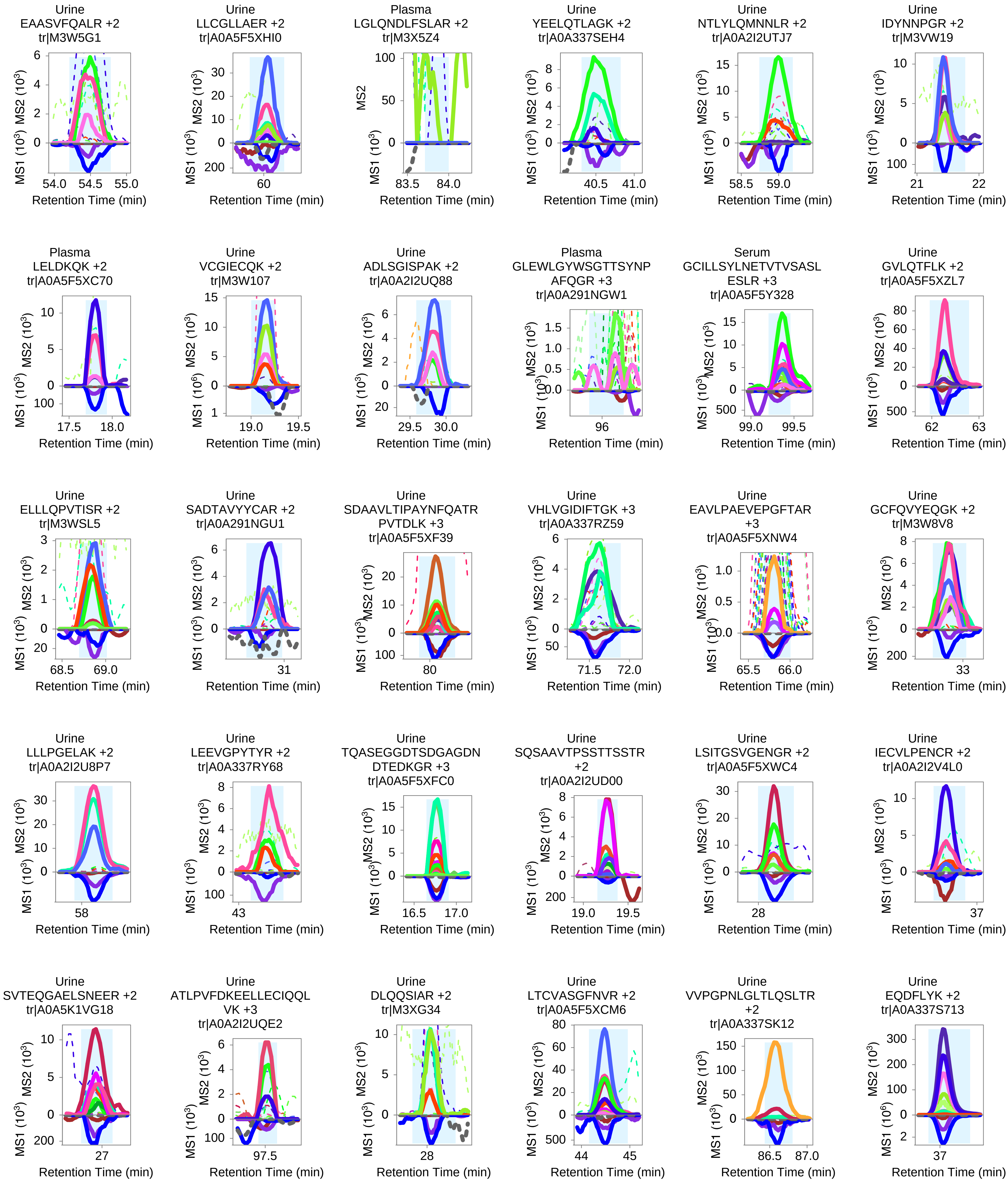
Urine
EAASVFQALR +2
tr|M3W5G1
MS1 (10^3) MS2 (10^3)
54.0 54.5 55.0
Retention Time (min)
Urine
LLCGLLAER +2
tr|A0A5F5XHI0
MS1 (10^3) MS2 (10^3)
60
Retention Time (min)
Plasma
LGLQNDLFSLAR +2
tr|M3X5Z4
MS1 (10^3) MS2
83.5 84.0
Retention Time (min)
Urine
YEELQTLAGK +2
tr|A0A337SEH4
MS1 (10^3) MS2 (10^3)
40.5 41.0
Retention Time (min)
Urine
NTLYLQMNNLR +2
tr|A0A2I2UTJ7
MS1 (10^3) MS2 (10^3)
58.5 59.0
Retention Time (min)
Urine
IDYNNPGR +2
tr|M3VW19
MS1 (10^3) MS2 (10^3)
21 22
Retention Time (min)
Plasma
LELDKQK +2
tr|A0A5F5XC70
MS1 (10^3) MS2 (10^3)
17.5 18.0
Retention Time (min)
Urine
VCGIECQK +2
tr|M3W107
MS1 (10^6) MS2 (10^3)
19.0 19.5
Retention Time (min)
Urine
ADLSGISPAK +2
tr|A0A2I2UQ88
MS1 (10^3) MS2 (10^3)
29.5 30.0
Retention Time (min)
Plasma
GLEWLGYWSGTTSYNP
AFQGR +3
tr|A0A291NGW1
MS1 (10^3) MS2 (10^3)
96
Retention Time (min)
Serum
GCILLSYLNETVTVSASL
ESLR +3
tr|A0A5F5Y328
MS1 (10^3) MS2 (10^3)
99.0 99.5
Retention Time (min)
Urine
GVLQTFLK +2
tr|A0A5F5XZL7
MS1 (10^3) MS2 (10^3)
62 63
Retention Time (min)
Urine
ELLLQPVTISR +2
tr|M3WSL5
MS1 (10^3) MS2 (10^3)
68.5 69.0
Retention Time (min)
Urine
SADTAVYYCAR +2
tr|A0A291NGU1
MS1 (10^3) MS2 (10^3)
31
Retention Time (min)
Urine
SDAAVLTIPAYNFQATR
PVTDLK +3
tr|A0A5F5XF39
MS1 (10^3) MS2 (10^3)
80
Retention Time (min)
Urine
VHLVGIDIFTGK +3
tr|A0A337RZ59
MS1 (10^3) MS2 (10^3)
71.5 72.0
Retention Time (min)
Urine
EAVLPAEVEPGFTAR
+3
tr|A0A5F5XNW4
MS1 (10^3) MS2 (10^3)
65.5 66.0
Retention Time (min)
Urine
GCFQVYEQGK +2
tr|M3W8V8
MS1 (10^3) MS2 (10^3)
33
Retention Time (min)
Urine
LLLPGELAK +2
tr|A0A2I2U8P7
MS1 (10^3) MS2 (10^3)
58
Retention Time (min)
Urine
LEEVGPYTYR +2
tr|A0A337RY68
MS1 (10^3) MS2 (10^3)
43
Retention Time (min)
Urine
TQASEGGDTSDGAGDN
DTEDKGR +3
tr|A0A5F5XFC0
MS1 (10^3) MS2 (10^3)
16.5 17.0
Retention Time (min)
Urine
SQSAAVTPSSTTSSTR
+2
tr|A0A2I2UD00
MS1 (10^3) MS2 (10^3)
19.0 19.5
Retention Time (min)
Urine
LSITGSVGENGR +2
tr|A0A5F5XWC4
MS1 (10^3) MS2 (10^3)
28
Retention Time (min)
Urine
IECVLPENCR +2
tr|A0A2I2V4L0
MS1 (10^3) MS2 (10^3)
37
Retention Time (min)
Urine
SVTEQGAELSNEER +2
tr|A0A5K1VG18
MS1 (10^3) MS2 (10^3)
27
Retention Time (min)
Urine
ATLPVFDKEELLECIQQL
VK +3
tr|A0A2I2UQE2
MS1 (10^3) MS2 (10^3)
97.5
Retention Time (min)
Urine
DLQQSIAR +2
tr|M3XG34
MS1 (10^3) MS2 (10^3)
28
Retention Time (min)
Urine
LTCVASGFNVR +2
tr|A0A5F5XCM6
MS1 (10^3) MS2 (10^3)
44 45
Retention Time (min)
Urine
VVPGPNLGLTLQSLTR
+2
tr|A0A337SK12
MS1 (10^3) MS2 (10^3)
86.5 87.0
Retention Time (min)
Urine
EQDFLYK +2
tr|A0A337S713
MS1 (10^6) MS2 (10^3)
37
Retention Time (min)

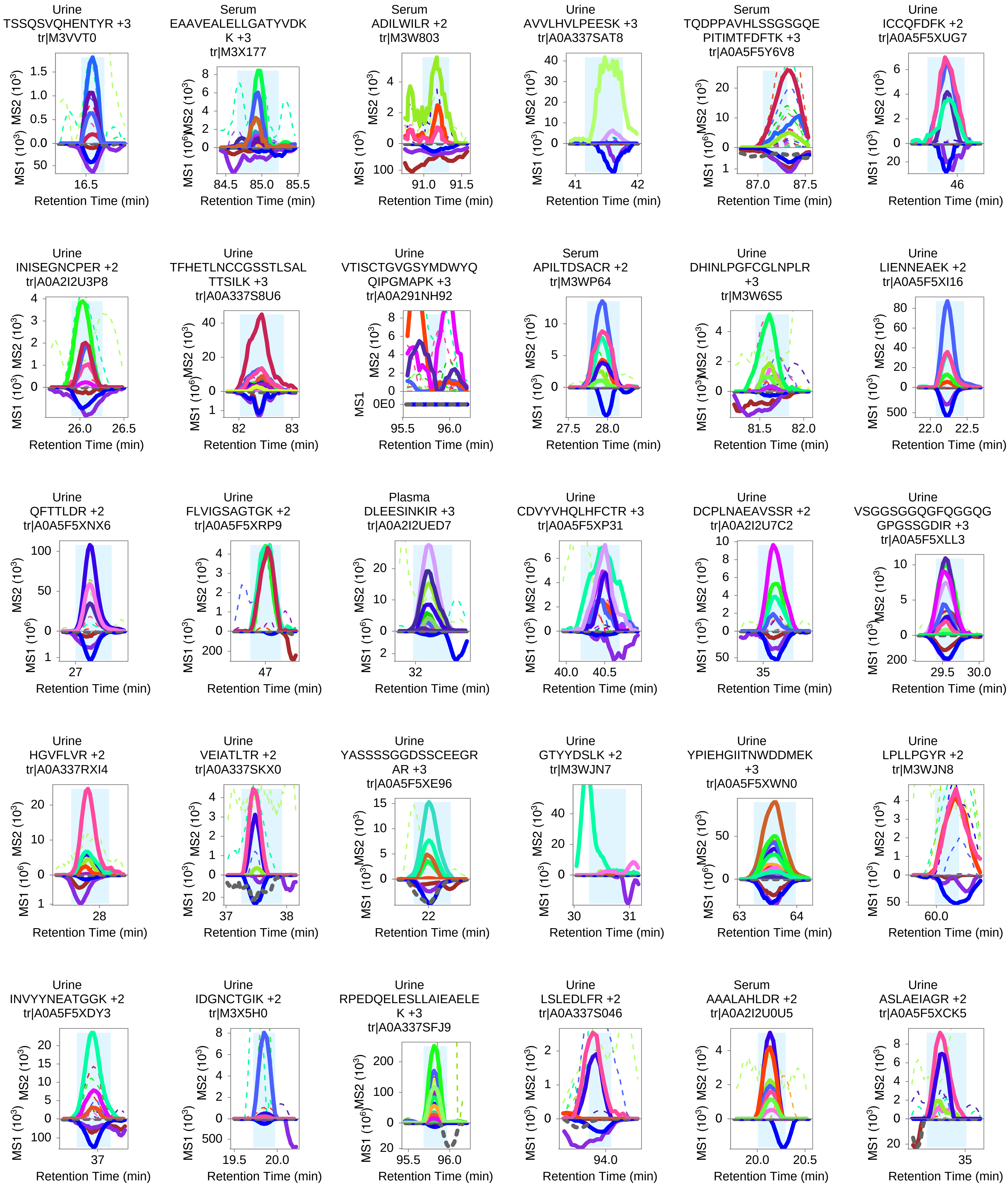

Urine
TSSQSVQHENTYR +3
tr|M3VVT0
MS1 (10³) MS2 (10³)
1.5
1.0
0.5
0.0
50
16.5
Retention Time (min)
Serum
EAAVEALELLGATYVDK
K +3
tr|M3X177
MS1 (10⁶) MS2 (10³)
8
6
4
2
0
84.5
85.0
85.5
Retention Time (min)
Serum
ADILWILR +2
tr|M3W803
MS1 (10³) MS2 (10³)
4
2
0
100
91.0
91.5
Retention Time (min)
Urine
AVVLHVLPEESK +3
tr|A0A337SAT8
MS1 (10³) MS2 (10³)
40
30
20
10
0
41
42
Retention Time (min)
Serum
TQDPPAVHLSSGSGQE
PITIMTFDFTK +3
tr|A0A5F5Y6V8
MS1 (10⁶) MS2 (10³)
20
10
0
1
87.0
87.5
Retention Time (min)
Urine
ICCQFDFK +2
tr|A0A5F5XUG7
MS1 (10³) MS2 (10³)
6
4
2
0
20
46
Retention Time (min)
Urine
INISEGNCPER +2
tr|A0A2I2U3P8
MS1 (10³) MS2 (10³)
4
3
2
1
0
26.0
26.5
Retention Time (min)
Urine
TFHETLNCCGSSTLSAL
TTSILK +3
tr|A0A337S8U6
MS1 (10⁶) MS2 (10³)
40
20
0
1
82
83
Retention Time (min)
Urine
VTISCTGVGSYMDWYQ
QIPGMAPK +3
tr|A0A291NH92
MS1 MS2 (10³)
8
6
4
2
0
0E0
95.5
96.0
Retention Time (min)
Serum
APILTDSACR +2
tr|M3WP64
MS1 (10³) MS2 (10³)
10
5
0
27.5
28.0
Retention Time (min)
Urine
DHINLPGFCGLNPLR
+3
tr|M3W6S5
MS1 (10³) MS2 (10³)
4
2
0
81.5
82.0
Retention Time (min)
Urine
LIENNEAEK +2
tr|A0A5F5XI16
MS1 (10³) MS2 (10³)
80
60
40
20
0
500
22.0
22.5
Retention Time (min)
Urine
QFTTLDR +2
tr|A0A5F5XNX6
MS1 (10⁶) MS2 (10³)
100
50
0
1
27
Retention Time (min)
Urine
FLVIGSAGTGK +2
tr|A0A5F5XRP9
MS1 (10³) MS2 (10³)
4
3
2
1
0
200
47
Retention Time (min)
Plasma
DLEESINKIR +3
tr|A0A2I2UED7
MS1 (10⁶) MS2 (10³)
20
10
0
2
32
Retention Time (min)
Urine
CDVYYVHQLHFCTR +3
tr|A0A5F5XP31
MS1 (10³) MS2 (10³)
6
4
2
0
40.0
40.5
Retention Time (min)
Urine
DCPLNAEAVSSR +2
tr|A0A2I2U7C2
MS1 (10³) MS2 (10³)
10
8
6
4
2
0
50
35
Retention Time (min)
Urine
VSGGSGGQGFQGGQG
GPGSSGDIR +3
tr|A0A5F5XLL3
MS1 (10³) MS2 (10³)
10
5
0
200
29.5
30.0
Retention Time (min)
Urine
HGVFLVR +2
tr|A0A337RXI4
MS1 (10⁶) MS2 (10³)
20
10
0
1
28
Retention Time (min)
Urine
VEIATLTR +2
tr|A0A337SKX0
MS1 (10³) MS2 (10³)
4
3
2
1
0
20
37
38
Retention Time (min)
Urine
YASSSSGGDSSCEEGR
AR +3
tr|A0A5F5XE96
MS1 (10³) MS2 (10³)
15
10
5
0
22
Retention Time (min)
Urine
GTYYDSLK +2
tr|M3WJN7
MS1 (10³) MS2 (10³)
40
20
0
30
31
Retention Time (min)
Urine
YPIEHGIITNWDDMEK
+3
tr|A0A5F5XWN0
MS1 (10⁶) MS2 (10³)
50
0
63
64
Retention Time (min)
Urine
LPLLPGYR +2
tr|M3WJN8
MS1 (10³) MS2 (10³)
4
3
2
1
0
50
60.0
Retention Time (min)
Urine
INVYYNEATGGK +2
tr|A0A5F5XDY3
MS1 (10³) MS2 (10³)
20
15
10
5
0
100
37
Retention Time (min)
Urine
IDGNCTGIK +2
tr|M3X5H0
MS1 (10³) MS2 (10³)
8
6
4
2
0
500
19.5
20.0
Retention Time (min)
Urine
RPEDQELESLLAIEAELE
K +3
tr|A0A337SFJ9
MS1 (10⁶) MS2 (10³)
200
100
0
20
95.5
96.0
Retention Time (min)
Urine
LSLEDLFR +2
tr|A0A337S046
MS1 (10³) MS2 (10³)
2
1
0
94.0
Retention Time (min)
Serum
AAALAHLDR +2
tr|A0A2I2U0U5
MS1 (10³) MS2 (10³)
4
2
0
20.0
20.5
Retention Time (min)
Urine
ASLAEIAGR +2
tr|A0A5F5XCK5
MS1 (10³) MS2 (10³)
8
6
4
2
0
20
35
Retention Time (min)

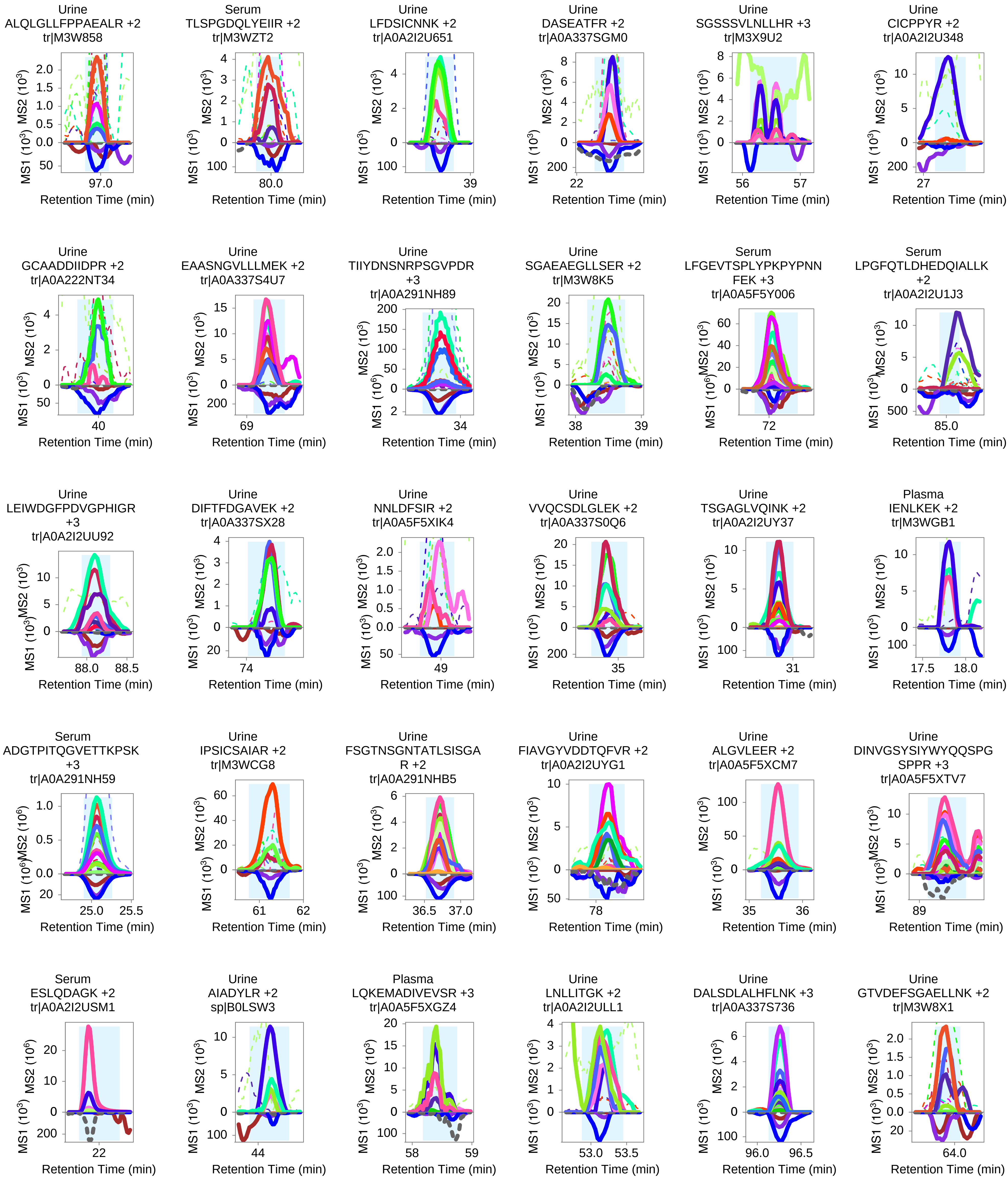

Urine
ALQLGLLFPPAEALR +2
tr|M3W858
Serum
TLSPGDQLYEIIR +2
tr|M3WZT2
Urine
LFDSICNNK +2
tr|A0A2I2U651
Urine
DASEATFR +2
tr|A0A337SGM0
Urine
SGSSSVLNLLHR +3
tr|M3X9U2
Urine
CICPPYR +2
tr|A0A2I2U348
Urine
GCAADDIIDPR +2
tr|A0A222NT34
Urine
EAASNGVLLLMEK +2
tr|A0A337S4U7
Urine
TIIYDNSNRPSGVPDR
+3
tr|A0A291NH89
Urine
SGAEAEGLLSER +2
tr|M3W8K5
Serum
LFGEVTSPLYPKPYPNN
FEK +3
tr|A0A5F5Y006
Serum
LPGFQTLDHEDQIALLK
+2
tr|A0A2I2U1J3
Urine
LEIWDGFPDVGPHIGR
+3
tr|A0A2I2UU92
Urine
DIFTFDGAVEK +2
tr|A0A337SX28
Urine
NNLDFSIR +2
tr|A0A5F5XIK4
Urine
VVQCSDLGLEK +2
tr|A0A337S0Q6
Urine
TSGAGLVQINK +2
tr|A0A2I2UY37
Plasma
IENLKEK +2
tr|M3WGB1
Serum
ADGTPITQGVETTKPSK
+3
tr|A0A291NH59
Urine
IPSICSAIAR +2
tr|M3WCG8
Urine
FSGTNSGNTATLSISGA
R +2
tr|A0A291NHB5
Urine
FIAVGYVDDTQFVR +2
tr|A0A2I2UYG1
Urine
ALGVLEER +2
tr|A0A5F5XCM7
Urine
DINVGSYSIYWYQQSPG
SPPR +3
tr|A0A5F5XTV7
Serum
ESLQDAGK +2
tr|A0A2I2USM1
Urine
AIADYLR +2
sp|B0LSW3
Plasma
LQKEMADIVEVSR +3
tr|A0A5F5XGZ4
Urine
LNLLITGK +2
tr|A0A2I2ULL1
Urine
DALSDLALHFLNK +3
tr|A0A337S736
Urine
GTVDEFSGAELLNK +2
tr|M3W8X1
MS2 (10³)
MS2 (10⁶)
MS1 (10³)
MS1 (10⁶)
Retention Time (min)

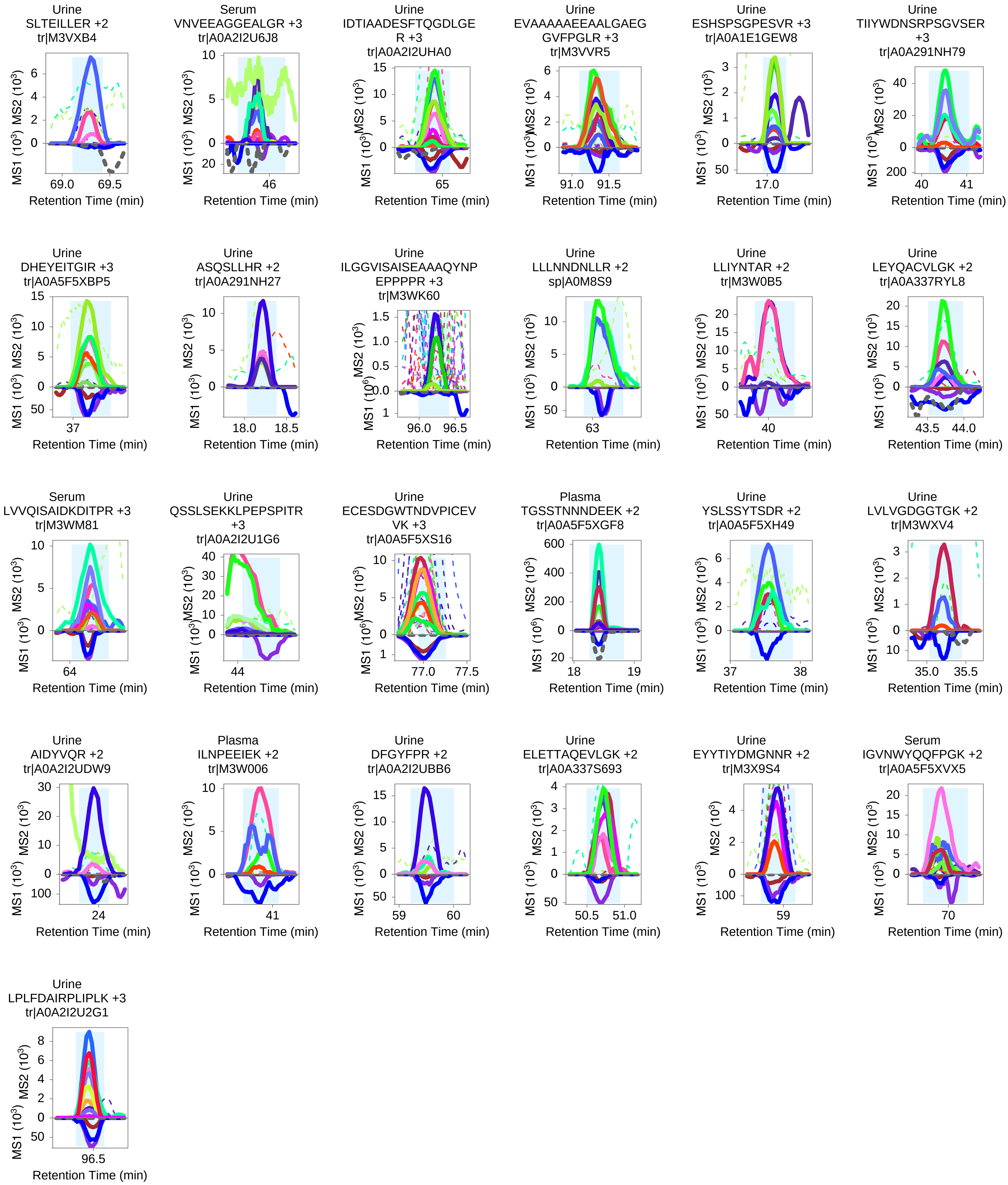
Urine
SLTEILLER +2
tr|M3VXB4
Serum
VNVEEAGGEALGR +3
tr|A0A2I2U6J8
Urine
IDTIAADESFTQGDLGE
R +3
tr|A0A2I2UHA0
Urine
EVAAAAAEEAALGAEG
GVFPGLR +3
tr|M3VVR5
Urine
ESHSPSGPESVR +3
tr|A0A1E1GEW8
Urine
TIIYWDNSRPSGVSER
+3
tr|A0A291NH79
Urine
DHEYEITGIR +3
tr|A0A5F5XBP5
Urine
ASQSLLHR +2
tr|A0A291NH27
Urine
ILGGVISAISEAAAQYNP
EPPPPR +3
tr|M3WK60
Urine
LLLNNDNLLR +2
sp|A0M8S9
Urine
LLIYNTAR +2
tr|M3W0B5
Urine
LEYQACVLGK +2
tr|A0A337RYL8
Serum
LVVQISAIDKDITPR +3
tr|M3WM81
Urine
QSSLSEKKLPEPSPITR
+3
tr|A0A2I2U1G6
Urine
ECESDGWTNDVPICEV
VK +3
tr|A0A5F5XS16
Plasma
TGSSTNNNDEEK +2
tr|A0A5F5XGF8
Urine
YSLSSYTSDR +2
tr|A0A5F5XH49
Urine
LVLVGDGGTGK +2
tr|M3WXV4
Urine
AIDYVQR +2
tr|A0A2I2UDW9
Plasma
ILNPEEIEK +2
tr|M3W006
Urine
DFGYFPR +2
tr|A0A2I2UBB6
Urine
ELETTAQEVLGK +2
tr|A0A337S693
Urine
EYYTIYDMGNNR +2
tr|M3X9S4
Serum
IGVNWYQQFPGK +2
tr|A0A5F5XVX5
Urine
LPLFDAIRPLIPLK +3
tr|A0A2I2U2G1
MS1 (10³) MS2 (10³)
Retention Time (min)
